# Thermostability of *In Vitro* Evolved *Bacillus subtilis* Lipase A: A Network and Dynamics Perspective

**DOI:** 10.1371/journal.pone.0102856

**Published:** 2014-08-14

**Authors:** Ashutosh Srivastava, Somdatta Sinha

**Affiliations:** 1 CSIR- Centre for Cellular and Molecular Biology, Hyderabad, India; 2 Indian Institute of Science Education and Research Mohali, S. A. S. Nagar, Manauli, India; University of Lethbridge, Canada

## Abstract

Proteins in thermophilic organisms remain stable and function optimally at high temperatures. Owing to their important applicability in many industrial processes, such thermostable proteins have been studied extensively, and several structural factors attributed to their enhanced stability. How these factors render the emergent property of thermostability to proteins, even in situations where no significant changes occur in their three-dimensional structures in comparison to their mesophilic counter-parts, has remained an intriguing question. In this study we treat Lipase A from *Bacillus subtilis* and its six thermostable mutants in a unified manner and address the problem with a combined complex network-based analysis and molecular dynamic studies to find commonality in their properties. The Protein Contact Networks (PCN) of the wild-type and six mutant Lipase A structures developed at a mesoscopic scale were analyzed at global network and local node (residue) level using network parameters and community structure analysis. The comparative PCN analysis of all proteins pointed towards important role of specific residues in the enhanced thermostability. Network analysis results were corroborated with finer-scale molecular dynamics simulations at both room and high temperatures. Our results show that this combined approach at two scales can uncover small but important changes in the local conformations that add up to stabilize the protein structure in thermostable mutants, even when overall conformation differences among them are negligible. Our analysis not only supports the experimentally determined stabilizing factors, but also unveils the important role of contacts, distributed throughout the protein, that lead to thermostability. We propose that this combined mesoscopic-network and fine-grained molecular dynamics approach is a convenient and useful scheme not only to study allosteric changes leading to protein stability in the face of negligible over-all conformational changes due to mutations, but also in other molecular networks where change in function does not accompany significant change in the network structure.

## Introduction

Retaining functional activity and structural integrity of the enzyme is a desirable quality for a number of proteins finding application in biotechnology, food processing and other commercial industries, where high temperature processes are used [1]. It is also important with regard to the insights that it might give on the overall stability of the proteins. Therefore, thermostability in proteins has been studied for a long time both experimentally as well as computationally [2]–[6]. Experimental approaches primarily focus on sequence comparison between the mesophilic and thermophilic proteins followed by mutating the mesophilic proteins in order to gain thermostability [2]. Computational studies involve structural comparison of the meso- and thermophilic proteins and correlating the differences to the stability of the proteins [3], [4]. Molecular dynamics simulations have also been employed to study the differences in the dynamics of these two types of proteins and correlating it to higher stability of the former [5], [6]. Thermostability is an emergent property of the protein that is imbibed through several structural factors that arise due to mutations of its mesophilic counter-part. Sequence and structure based comparison of the known thermophilic homologs of the mesophilic proteins has shed some light on the factors that might be contributing to the higher stability of thermophilic proteins.

Although several factors can lead to stability of the proteins, some properties are found to be consistently correlated to the higher stability of proteins. These include, higher number of hydrogen bonds [7]–[9] and salt bridges [10]–[12], stabilization of secondary structures [13], introduction of disulphide bonds [14], higher number of Proline residues [15], [16], higher polar surface area [8], [17], shortening and stabilization of loops [7], [12] and increased buried surface area after oligomerization [18]. Recently, surrounding hydrophobicity has also been found to be an important property distinguishing the thermophilic from mesophilic proteins [19]. However, there are certain confounding factors in such comparisons, and the differences observed may not be completely attributable to thermostability. Some of these differences might be present due to phylogenetic differences and thus be representative of just different ancestry rather than physico-chemical implications [4].

In this regard, “directed evolution” has been proven to be successful in studying and designing enzymes with specific properties without being confounded by the phylogenetic differences inherent in the comparison of the natural homologs. This involves using a series of experimental techniques for mutation and recombination of the protein and selection on the basis of a certain property [20]. Directed evolution has been used extensively to achieve substantial thermostability in proteins. The prominent examples being p53 [21], p-nitrobenzyl esterase [22], xylanase [23], subtilisin E [24], β-glucosidase [25] and *Bacillus subtilis* Lipase A [26]–[28]. By studying the thermostable mutants produced by directed evolution one can study the structural features leading to thermostability in isolation as the protocol dictates only the selection of those mutations that contribute to thermostability.

Usually, while studying the effect of mutations on protein functions, only the areas in the vicinity of mutation are analyzed in detail. However, studies have shown that small conformational changes occurring in one region of the protein can have an effect in a region quite far from the point of perturbation without affecting the functionality [29]. Hence it has become pertinent to study the effects of mutations on the protein as a whole, rather than concentrating only on the regions of perturbation, because multiple small conformational changes may lead to an overall effect on the functionality of the protein. This becomes all the more evident when the overall conformational change occurring in the proteins, due to such distributed mutations, is small.

Working on this premise, in the present study, we have chosen the wild-type and six *in-vitro* evolved thermostable mutants of *Bacillus subtilis* Lipase A protein, in which increasing thermostability has emerged due to distributed mutations (ranging from 2 to 12 positions) without causing any significant difference in their three-dimensional structures [26]–[28]. We have analyzed the wild type (WT) *Bacillus subtilis* Lipase A structure, and the factors leading to thermostability in its six thermostable mutant structures (Table 1), by modeling them using a combination of network-based approach (Protein Contact Networks or PCN) [30] and molecular dynamics (MD) studies. Recently network based approach has been used extensively for studying the structure function relationship in proteins. Protein Contact Networks have been used to predict residues crucial for signaling within the protein [31], to study the allosteric communication in the proteins [32], to understand the folding kinetics [33], etc. However, in the present work we have attempted to utilize this approach to study small conformational changes occurring in the protein due to mutations, by analyzing the contact patterns and their positional information in all the six mutants and the WT, with respect to their structural stability and functionality at higher temperatures. Molecular Dynamics has been employed here to support the results drawn from the network analysis, as well as, to gain insights into the effect of mutations on the dynamics of the protein, and more specifically, the role that it might be playing in thermostability caused by the mutations. Our results indicate that this combined meso-scale network analysis and fine-scale dynamic simulation approach can be both useful and important in understanding the key factors influencing the structural correlates and allosteric information transfer in rendering thermostability in these proteins.

**Table 1:** Structures used for the analysis.

Name	PDB ID	Mutations	Resolution (Å)	Optimum Temperature (°C)	Reference
WT	1I6W	Wild Type	1.50	35	34
DM	1T4M	N166Y, A132D	2.00	a	26
TM	1T2N	N166Y, A132D, L114P	1.80	45	26
1–17A4	3D2A	N166Y, A132D, L114P, I157M	1.73	a	27
2D9	3D2B	N166Y, A132D, L114P, I157M, F17S, N89Y	1.95	50	27
4D3	3D2C	N166Y, A132D, L114P, I157M, F17S, N89Y, A15S, A20E, G111D	2.18	55	27
6B	3QMM	N166Y, A132D, L114P, I157M, F17S, N89Y, A15S, A20E, G111D, M134E, M137P, S163P	1.89	65	28

a Optimum Temperatures not available.

## Results

**Figure 1:**
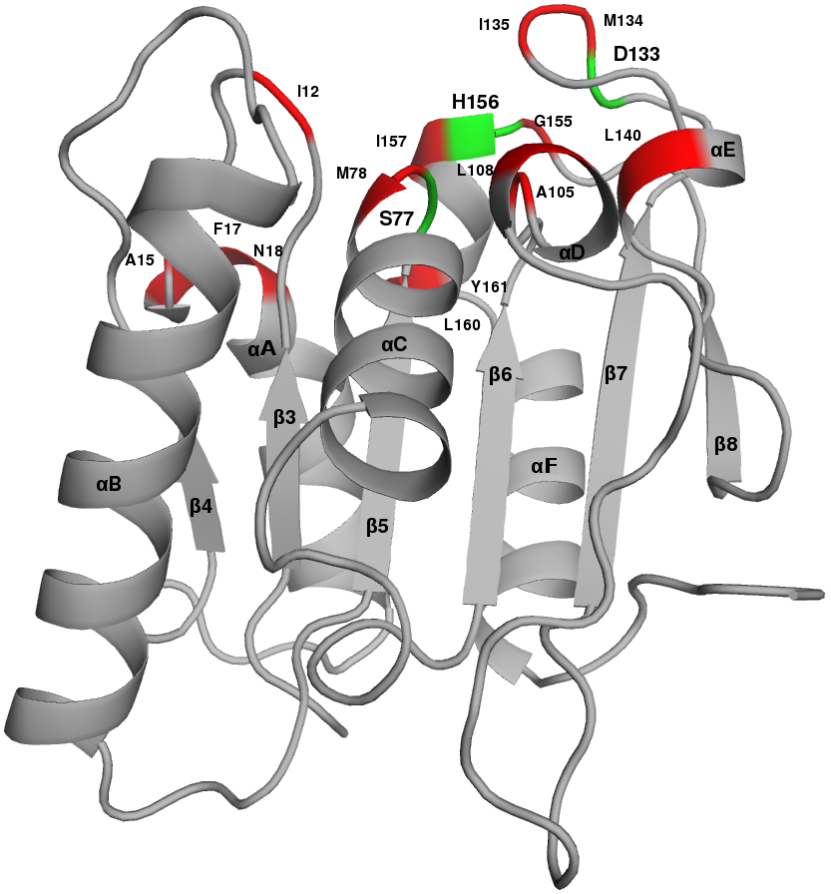
*Bacillus subtilis* Lipase A structure. The catalytic triad residues are shown in Green, and active site residues are shown in Red. The secondary structures (α-helices and β-strands) are labeled.

*Bacillus subtilis* Lipase A is a globular protein consisting of six β-strands in a parallel β-sheet surrounded by two α-helices on one side and three on the other [34] (Figure 1). The catalytic site of the protein is composed of three residues S77, D133 and H156. However, other nearby residues also participate in the overall function of the protein (Figure 1). The crystal structures [26]–[28] of six mutants of Lipase A generated from the wild type (WT), having increased thermostablity, used in this study are listed in Table 1.

The WT and the mutant structures were aligned (Figure S1) and the cross structure Root Mean Square Deviation (RMSD) between them was determined (Table 2). RMSD provides an average estimate of the overall structural variation between the molecules, and here the cross-structure RMSD ranges between 0.18–0.39 Å. This low RMSD signifies the minor changes that occur between the structures on acquiring 2 to 12 mutations. However, these nominal changes in the conformation and dynamics associated with them are responsible for the remarkable variation observed in the thermostability of the mutants. Since structural changes are quite small, inferences based on RMSD values alone are not informative in explaining the increased thermostability of the mutants. Hence we have utilized a network-based approach to quantify the small conformational changes occurring throughout the protein using simple assumptions.

**Table 2:** Cross structure RMSD values (Å) between mutants and WT.

	WT	DM	TM	1–17A4	2D9	4D3	6B
**WT**	0	0.344	0.374	0.319	0.326	0.304	0.393
**DM**		0	**0.181**	**0.394**	0.347	0.295	**0.181**
**TM**			0	0.385	0.35	0.289	0.361
**1–17A4**				0	0.291	0.327	0.314
**2D9**					0	0.345	0.352
**4D3**						0	0.315
**6B**							0

Values in bold show the lowest and the highest RMSD values observed.

### Network analysis of the WT and mutant protein structures

Six network parameters, namely, Degree, Betweenness Centrality (BC), Closeness Centrality, Clustering Coefficient, Shortest Path and Modularity were calculated for the Protein Contact Networks (PCNs) constructed from all seven structures (see Methods). The global (average) network parameters show very little difference among the structures (Table S1), and no correlation was found between the average network parameters and the temperature at which the mutant proteins were functional. This supports the diminutive changes in the overall structures as shown by the small cross-structure RMSD values in Table 2. To understand the role of small conformational changes occurring due to the mutations, the variations in local, residue-specific network parameters were studied in all seven PCNs of the WT and mutants of Lipase A.

#### Degree

**Figure 2:**
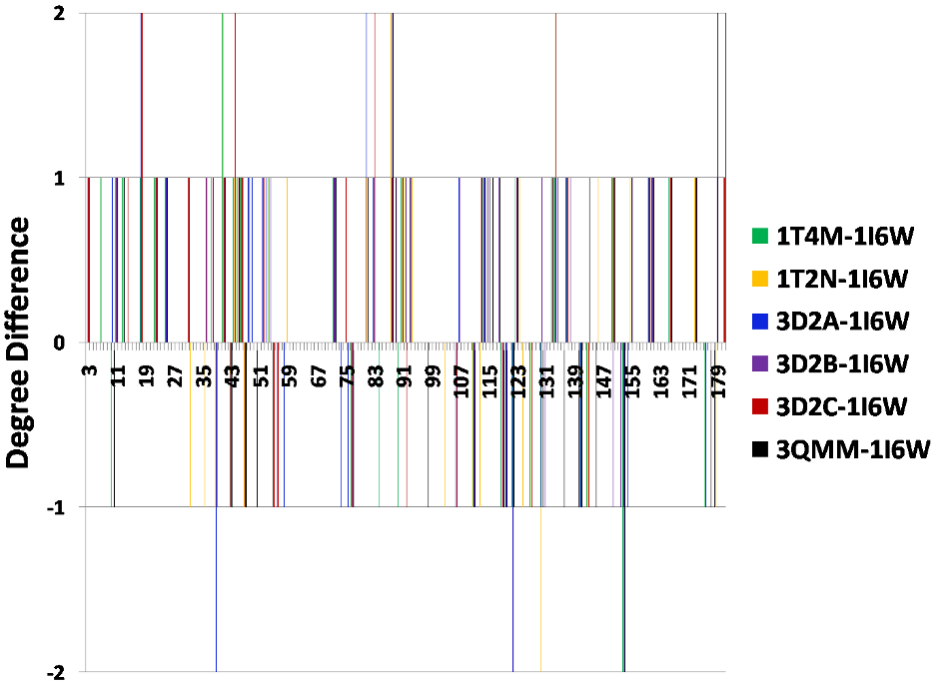
Residue-wise difference between the degree values of the mutants and WT.

On an average, 26% of the residues showed any change in degree in these PCNs (range: 23% (3D2A)–28% (1T2N and 3QMM)). Figure 2 shows the difference between the degree of each residue in the mutants and WT, and the maximum change observed (increase or decrease) was 2. The active site residues, N18 and M134, show increase in degree by 2 in four out of six mutants. Other non-active site residues showing increase of 2 are F41, K44, A81, T83, K88, N179, and N181. Of these, K44 and A81 are directly connected to the active site residues, N179 and N181 are C-terminal residues, and K88 lies close to a mutated residue N89Y. Thus these residues are responsible for the changes indirectly. As will be shown later, all these allosteric residues are involved in making new contacts in the mutants that can lead to small conformational changes, which can be correlated to the overall enhanced stability of the structures.

#### Betweenness Centrality (BC)

**Figure 3:**
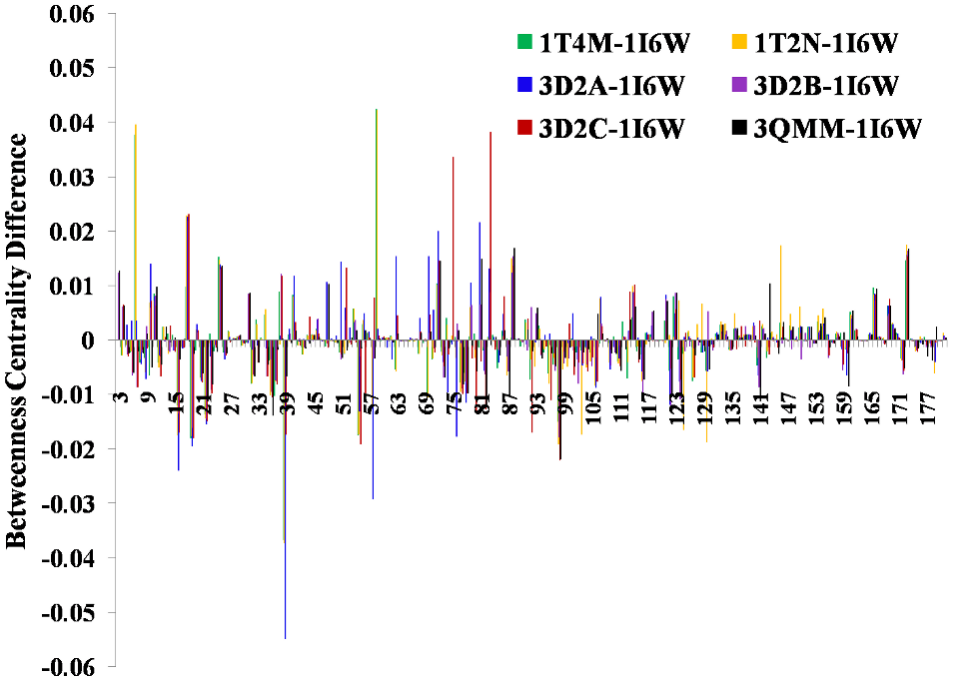
Residue-wise difference between then BC values of the mutants and WT.

High BC signifies that the node resides on several shortest paths in a network, and hence is crucial for communication across the different modules of the network. Significantly high BC (z-score>2.5) of all the residues in each PCN are listed in Table S2. The three hydrophobic residues (V100, L102, L159) in the core of the proteins consistently show high BC. The residue-wise differences in the BC between mutants and WT are shown in the Figure 3. Ten residues from each mutant showing highest positive and negative differences are listed in Table S3. The contacts formed and lost in the mutants are crucial in the overall connectivity of the protein network, which can also be inferred from the centrality value of these residues. The role of contact patterns in the mutants and their influence on the BC is discussed in the next section. Closeness Centrality measure of the residues do not show appreciable difference between the WT and mutants.

#### Clustering Coefficient (CC)

**Figure 4:**
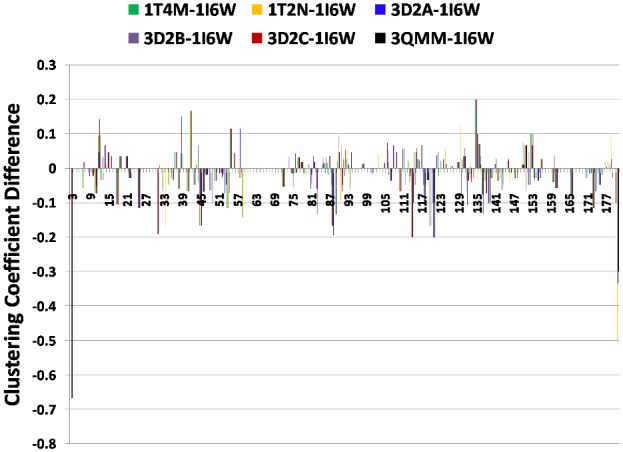
Residue-wise difference between the CC values of the mutants and WT.

CC measures the cliquishness of the neighborhood of a node [35]. Residues with high clustering coefficient (z-score>2.0) were determined for all the PCNs. The three hydrophilic non-active site residues, D34, N/Y89, and N120, that are solvent-exposed in all the structures, have consistently high CC in all PCNs (Table S4). Residues I135 and G153 that are close to the catalytic triad residues D133 and H156, show increase in CC in all the mutants (Table S5). Residues present near the active site - I12, G14, A/S15, N106, L108, S131 and V136 - also show increase in the clustering coefficient in different mutants (Figure 4). This signifies increase in the interconnectedness of the region near the active site, which can contribute to further stabilization of the region [36]. A substantial decrease is observed in the clustering coefficients of the terminal residues due to addition of new neighbors resulting from the new contacts formed in mutants (keeping the older contacts among these neighbors the same).

Analysis of residue-specific network parameters offers several important clues towards the residues that may have a role to play in governing structural stability under thermal stress in the mutant proteins. To elaborate these, in the next section, we analyze the contact patterns in the PCNs of WT and the mutant proteins. One important feature that is revealed through this analysis is that most changes in network parameters occur at the residues that are not part of the active site of the proteins, indicating the specific role of allosteric sites in these thermostable proteins whose overall conformations do not change due to the mutations.

### Analysis of Contacts in PCNs

**Figure 5:**
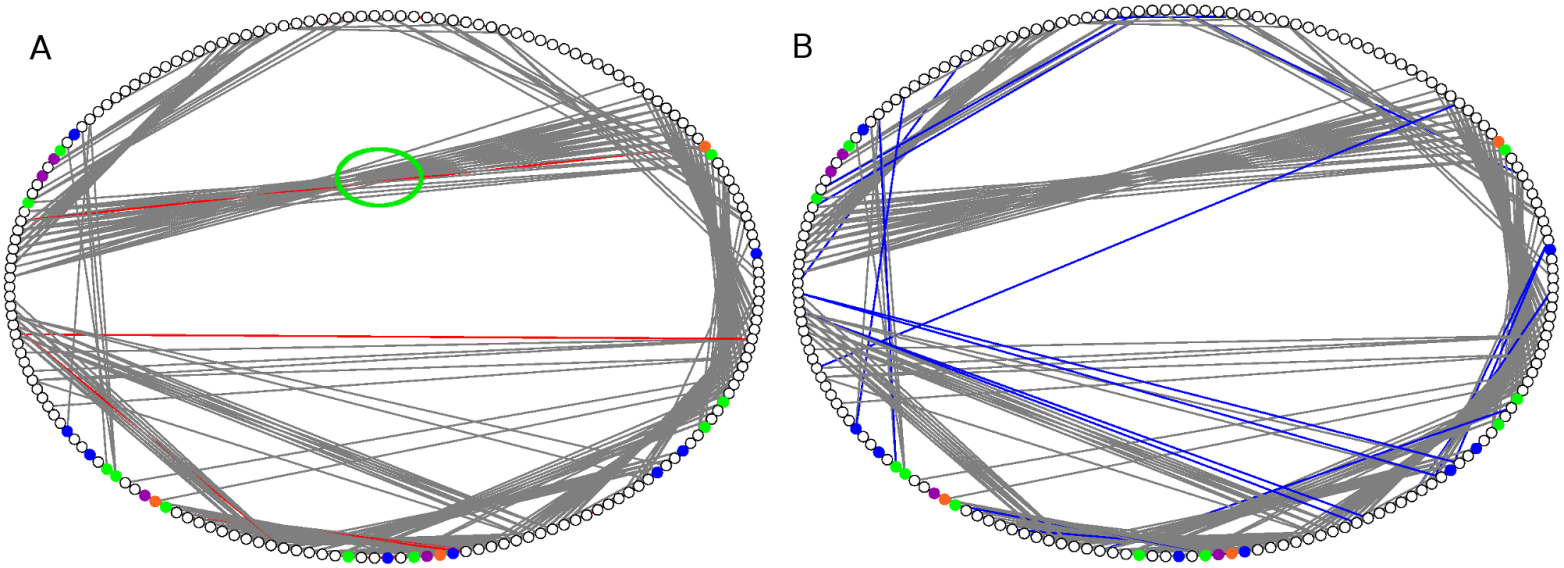
Ring Graph representation of contacts lost and made. Ring Graph representation showing A) the contacts lost (Red) and B) the contacts made (Blue) in the mutant 6B. The nodes are color-coded. Active Site residues have been shown in Green, Catalytic triad residues in Orange, Mutated residues in Blue, and Active site Mutated residues in Purple. The lost contacts have been shown in the PCN of WT.

Since there is little overall conformational change among the structures (Table 2), and the local network properties point towards important roles of specific residues at certain allosteric sites of the mutants, along with analysis of all mutants, we also performed an in depth analysis of changes in contact patterns in the most thermostable mutant (3QMM or 6B) in comparison to the WT. The PCN representation is particularly useful for highlighting the contacts among residues in the three-dimensional structure space. The ring graph representation of the two PCNs (WT and 6B shown in Figure 5) can easily demonstrate the changes in their contact patterns. The edges across the circle represent the long-range contacts in the protein tertiary structures. Consequently the contacts between β-strands in a β sheet are often visible as long edges in the centre of the circle (green circle in Figure 5A). Helices contain short-range contacts (n to n+4) and hence are visible along the circle boundary. However inter-helical contacts in the tertiary structures are visible as long-range contacts in the centre of the ring graph. In a PCN, the occurrence of a new contact signifies decrease and loss of contact indicates an increase in the distance (below the threshold) between two residues. The contacts lost (Red) and made (Blue) in the mutant 6B have been indicated in the ring graphs of WT and 6B respectively (Figure 5A and B). The figure shows that, compared to the WT, the long-range contacts made in the mutant are more than the long-range contacts lost. The ring graphs showing the contacts lost and made in the other five mutants are shown in Figure S2. Analysis of the new contacts made and lost also revealed the fact that most changes in contacts in the mutants are at the allosteric sites and do not involve the active site residues.

**Figure 6:**
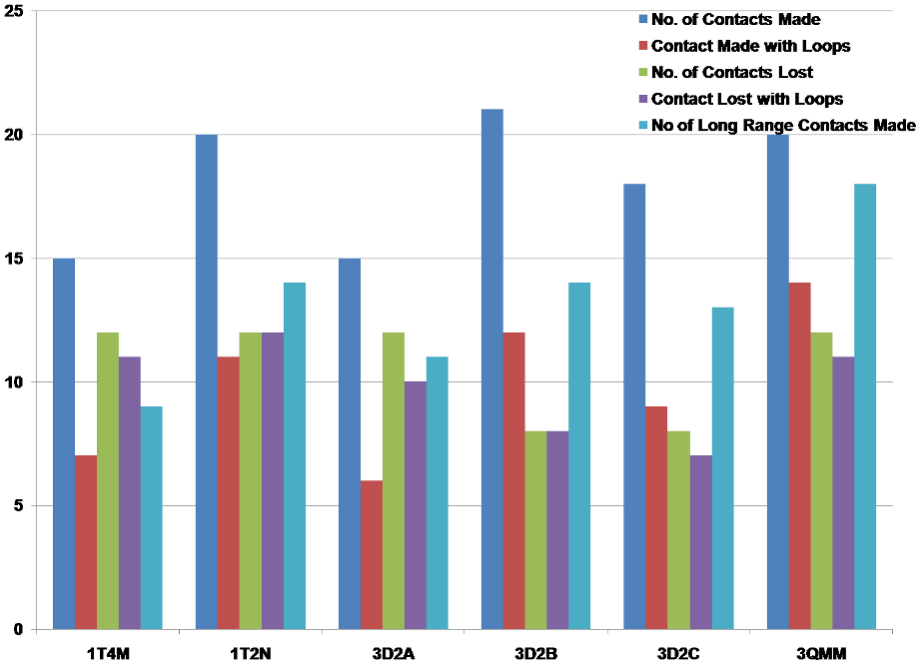
Number of contacts made and lost in the mutants. Contacts made (Blue) and lost (Green) in all the mutants as compared to the WT. Red bars show the number of contacts made with loops and purple bars show the number of contacts lost in loops. Cyan bars show the number of long range contacts made in mutants.

Figure 6 shows the number of new contacts made and lost, compared to the WT. The figure clearly indicates that the number of new contacts made is consistently higher than the number of contacts lost in all mutants (details given in Table S6 and S7). Among all the contacts made and lost mentioned in the Tables S6 and S7, there were a total of 36 unique contacts made and 27 unique contacts lost in all the mutants combined. Of these, 67% (24 out of 36) of the new contacts made are long-range contacts, and roughly half (52% or 14 out of 27) of the contacts lost are short range, in the mutated structures. Additionally, Figure 6 also shows that almost all contacts lost in the six mutant structures involve the loop region residues. This clearly suggests considerable reorganization in the loop regions of the thermostable mutant structures. An interesting point to note here (which is clear in Figure 5) is that the contacts are made and lost throughout the structure, and not necessarily at the sites of mutation or at the functional residues. This suggests that the conformational changes are distributed throughout the structure, and contribute to the resultant thermostability. This trend is also seen in PCNs constructed with higher and lower cut-offs (see Methods).

**Figure 7:**
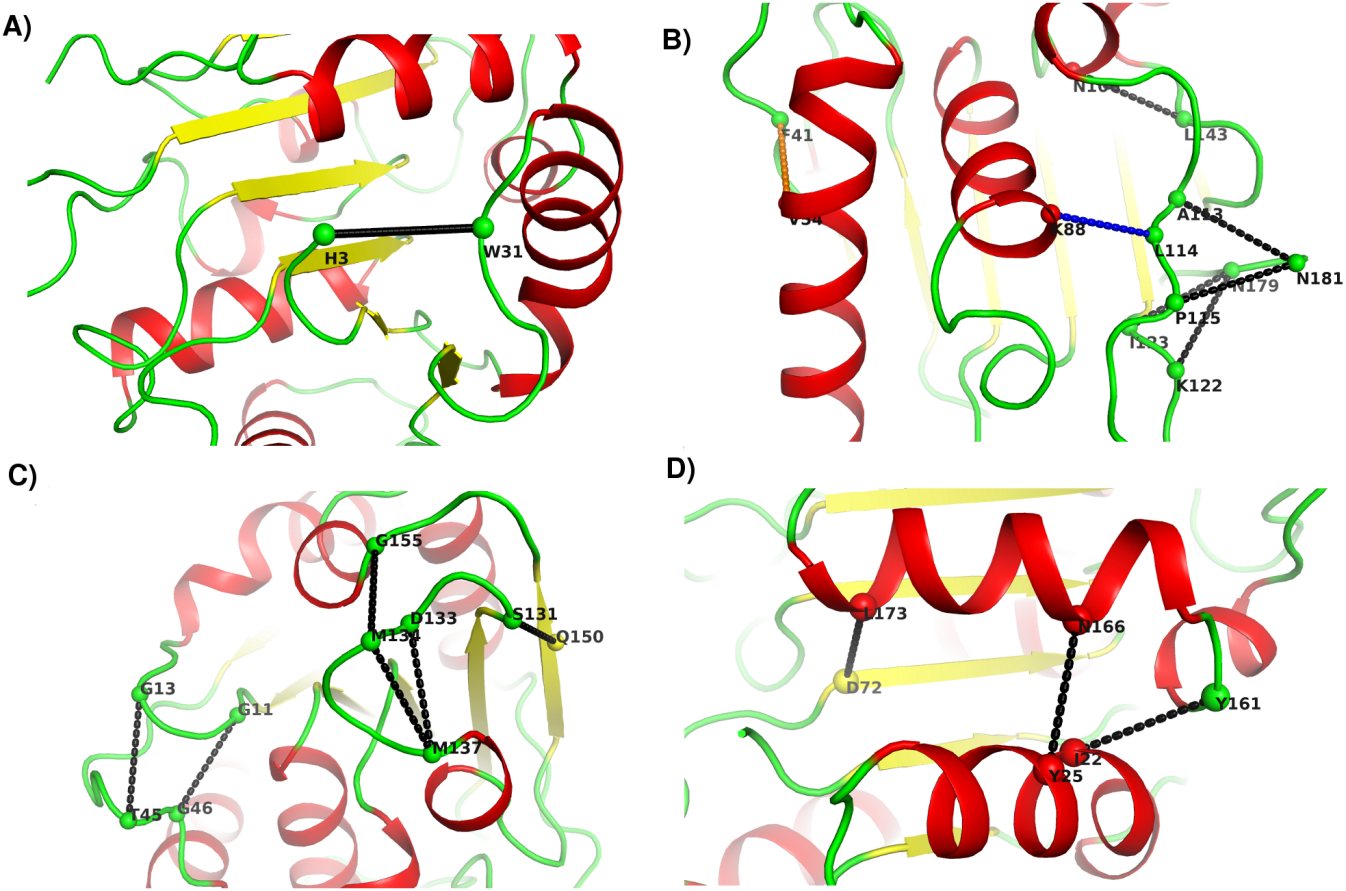
New contacts mapped on the structure of Lipase. Contacts made in the PCN of 6B have been shown on the tertiary structure of the Lipase. A) Contact between W31 and H3 stabilizing the N terminus; B) Contacts stabilizing the C-terminus (shown in Black) and loop (shown in Blue and Orange); C) Contacts near active site (active site residues given in Table 1) and D) Contacts joining two secondary structures.

In order to understand the role of these new contacts on the overall stability of the mutants, the location of these contacts is shown in the three dimensional structure (Figure 7). These new contacts in the PCNs are obtained using a purely geometric criterion, and not on the basis of the physico-chemical interactions. Below we categorize these contacts based on their likely effects on the structural stability and function of the proteins.

#### (a) Termini stabilizing contacts

Figure 7A and 7B show the specific contacts (in black) between residues (31 & 3, and 181 & 113/115) that help stabilize the N- and C-termini of the mutant protein 6B. All the mutants with L114P mutation (1T2N, 3D2A, 3D2B, 3D2C and 3QMM) show this terminal stabilizing contact (Figure 7B). This also corroborates the structural studies that attribute L114P mutation to the stability of C-terminus of the Lipase A structure [26]. Two new contacts observed between the residues N179 and K122, I123 in some mutant PCNs correspond to a salt bridge that has been described in a previous study between N181 and K122 as a result of the L114P mutation [26].

#### (b) Loop stabilizing contacts

About 53% of the total new contacts made (19 out of 36) involve one of the residues from a loop region, and 89% (24 out of 27) of the contacts lost are from the loop regions in the mutants. These flexible regions (loops and turns) of the proteins are vulnerable during the unfolding process. Both contacts made and lost in these regions lead to the reorganization of the loop regions that, along with the new contacts made as described in earlier and later sections, might be contributing to the overall stability of the proteins. For the mutant 6B PCN, 14 out of 20 new contacts made have at least one residue from the loop region, and 11 out of 12 contacts lost are from the loop regions. Similar trend is seen in all mutants (Figure 6).

Figure 7B shows that a new contact (blue) between P114 and K88 (connecting the longest loop and the C-terminus of helix αC) occurs in all the five structures with L114P mutation. This, along with the termini stabilizing contacts between loop and the C-terminus residues, contributes to the overall stability of the protein (Figure 7A & B). The contact (54 to 41) connecting the helix αB to a loop between β4 and αB (Figure 7B: orange contact), and the contact between residues 143 and 106 (connecting the loop between αE and β8 to the 3_10_ helix αD) – also contribute to the reduction in the flexibility of the loop regions as is shown later in molecular dynamics studies (see section on RMSF).

#### (c) Contacts leading to active site rigidity

Rigidity of the region surrounding the active site in mutant proteins was suggested to be responsible for thermostability in a previous experimental and dynamic simulation study [36]. Our analysis shows new contacts between G46-G11 and T45-G13 (connecting two loops in Figure 7C) have residues 11 and 13 positioned close to two mutated residues A15S and F17S and the active site residues I12 and N18. Four mutated residues (A132 (D), M134 (E), M137 (P) and I157 (M) near the catalytic triad) and residues adjacent to them show four new contacts (137–133, 137–134, 150–131 and 155–134) in the mutant PCNs. Such anchoring of the flexible regions and presence of new contacts can lead to rigidity in the region surrounding the active site in the mutant protein. As mentioned earlier, the residues in this region also show an increase in their clustering coefficient, which indicates development of rigidity in the region.

#### (d) Contacts connecting regular secondary structures

Figure 7D shows two new contacts between helix αF and helix αA (166-25 and 161-22) present in all mutants but absent in the wild type. These have been shown in an earlier structural study on two mutants [26] as parallel-displaced pi-stacking and gain of a hydrogen bond. Another new contact L173-D72 (connecting helix αF and β5) is present in all the mutants. Thus a simple meso-scale network representation can easily give a clear idea of the new contacts made for comparison between many proteins.

These local effects of contacts made/lost are also visible in the network parameters at the residue-level. Increase in degree, clustering coefficient, and Betweenness Centrality are observed for most of the allosteric residues discussed above, which are implicated to contribute to reduction of flexibility and increase in rigidity of mutant structures with little change in overall conformation. Some of these have been discussed in Text S1.

### Betweenness Centrality and new Contacts

**Figure 8:**
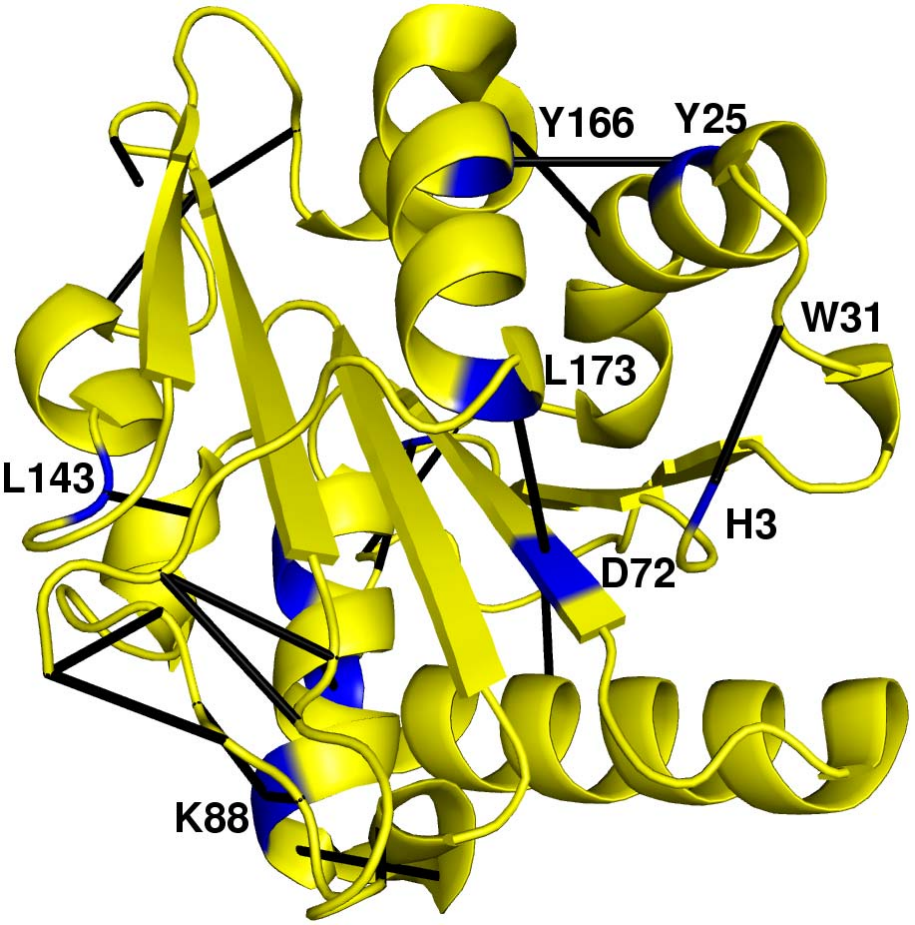
Ten residues showing highest increase in BC along with the contacts made mapped on the WT structure. Residues are shown in Blue and the Black lines represent the new contacts made in the 6B mutant as compared to WT PCN.

High BC of a residue is crucial for communication across different modules of the PCN, as it resides on several shortest paths in a network. Figure 8 shows the structure of WT with the ten residues with highest increase in BC (z-score 2.74–3.21, Table S2 and Text S1) along with the new contacts made in 6B. Residue D72 (in central β5) has high Betweenness Centrality (z-score 2.74), and it also makes a new contact with residue L173 (in outer helix αF) in all the mutants. This contact increases the number of shortest paths passing through these nodes thus increasing their BC. Similar increase in BC is observed in both Y25 and Y166, and they make a new contact between them in all the mutants. Other residues showing increase in the BC are N18, H3 and K88, which also make new contacts in the mutants. This suggests that the contacts formed in the mutants are crucial in the overall connectivity and information transfer among residues in the mutant protein network.

### Community Structure Analysis of the PCNs

Modularity is defined by groups of nodes within the network that are more connected among themselves compared to the other nodes. PCNs have been shown to possess modules, which correspond to the functional regions of the protein, and have been implicated in efficient transfer of information between different domains of the protein [37]. Even though the WT and mutant proteins do not show much difference among their three dimensional structures, an analysis of the contacts points towards addition of new contacts and loss of some others due to specific mutations, that can influence information transfer. To assess if these changes, which may not induce overall structural changes, can lead to reorganization of the modules in the structures, we determined the community structures for all seven PCNs. This analysis gives five to six communities in all the structures, and the residues belonging to different communities are listed in Table S8.

**Figure 9:**
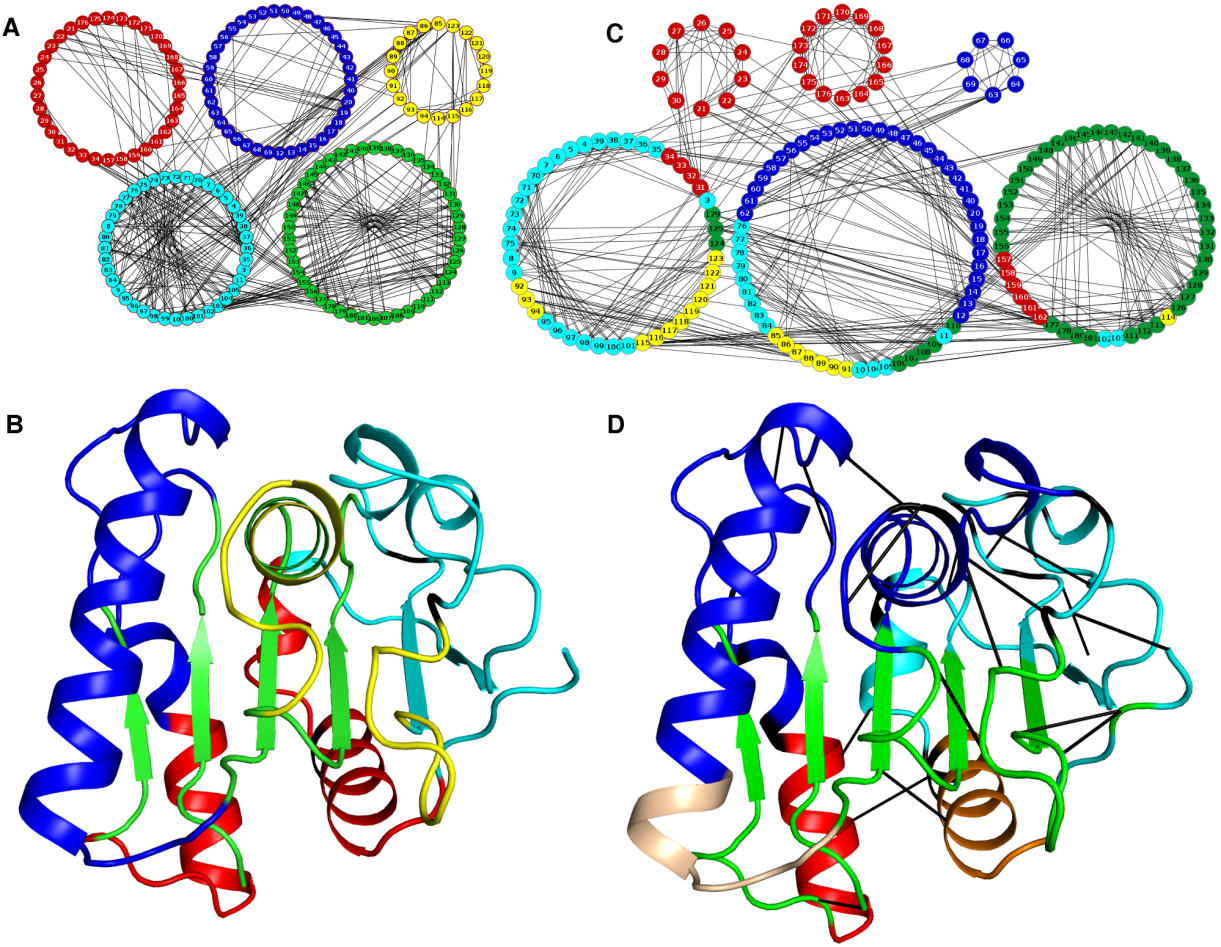
Community structure in the WT and 6B mutant. A) Community structure in WT showing five communities in five different colors. B) Five communities mapped on the tertiary structure of WT. C) Community structure in 6B showing six different communities. The colors of the nodes have been shown according to membership in WT in order to indicate the reorganization of the communities. D) Six communities mapped on the tertiary structure of 6B. The Black lines represent the new contacts made in the 6B as compared to WT.

Figure 9 shows the structures of the WT and 6B and the corresponding communities with residues colour-coded according to the communities. The communities in the mutant PCN show extensive reorganization in membership of nodes compared to the WT (Figure 9A and 9C and Table S8). For example, Community 1 in WT (Residues 21–34 and 157–176, red nodes in Figure 9A and 9B) is split in all the mutants. The probable cause for this split is the first mutation N166Y in the WT structure that separates the single community involving the two secondary structures (helices αA and αF) into two (red and orange residues) in Figure 9D. It is interesting to note that there are two new contacts that have been shown to occur between these two helices (αF and αA) in all mutants. This has been earlier [26] implicated to increase the interaction between them, but our community analysis shows that these residues now belong to two different communities. This indicates that the new contacts may be helping predominantly in stabilizing the two helices, but have reduced communication between them. Interestingly, Community 3 (yellow) in WT, containing residues of the longest loop (114–123), merges with a larger community having the central β sheet (green residues in Figure 9) in the mutant. This is indicative of an increase in communication between the loop and the β sheet in the mutant due to changes in the contact patterns in nearby regions. This is reminiscent of the L114P mutation and the loop stabilizing contacts. One of the most important observations here is that the community (green) containing nodes from central beta sheet expands in the mutant. This inclusion of greater number of nodes in the central community shows that the edge density within the core structure has increased leading to strengthening of the core structure, thereby increasing the overall stability of the structure.

The PCN analyses of the WT and mutant structures offer several important clues to the possible finer structural factors, involving residues from non-active site regions, which can lead to overall stability that aids in thermostability and retention of functionality in *Bacillus subtilis* Lipase A.

### Analysis of Molecular Dynamics Simulations

In order to understand the role played by the entire molecule in the thermal stability and the factors responsible for it, we performed equilibrium MD simulations on the WT and two mutant structures (2D9 and 6B) having different numbers of mutations and exhibiting functionality at increasing temperature (see Table 1). Earlier biophysical and MD (20 ns) studies [36] have focused only on the active site geometry and studied the rigidity and dynamics in the 6B mutant. In our study we have monitored all the new contacts (near and far from the active site) observed in the two mutants in molecular dynamics simulations in order to see the stabilizing role of these contacts in overall protein dynamics.

**Figure 10:**
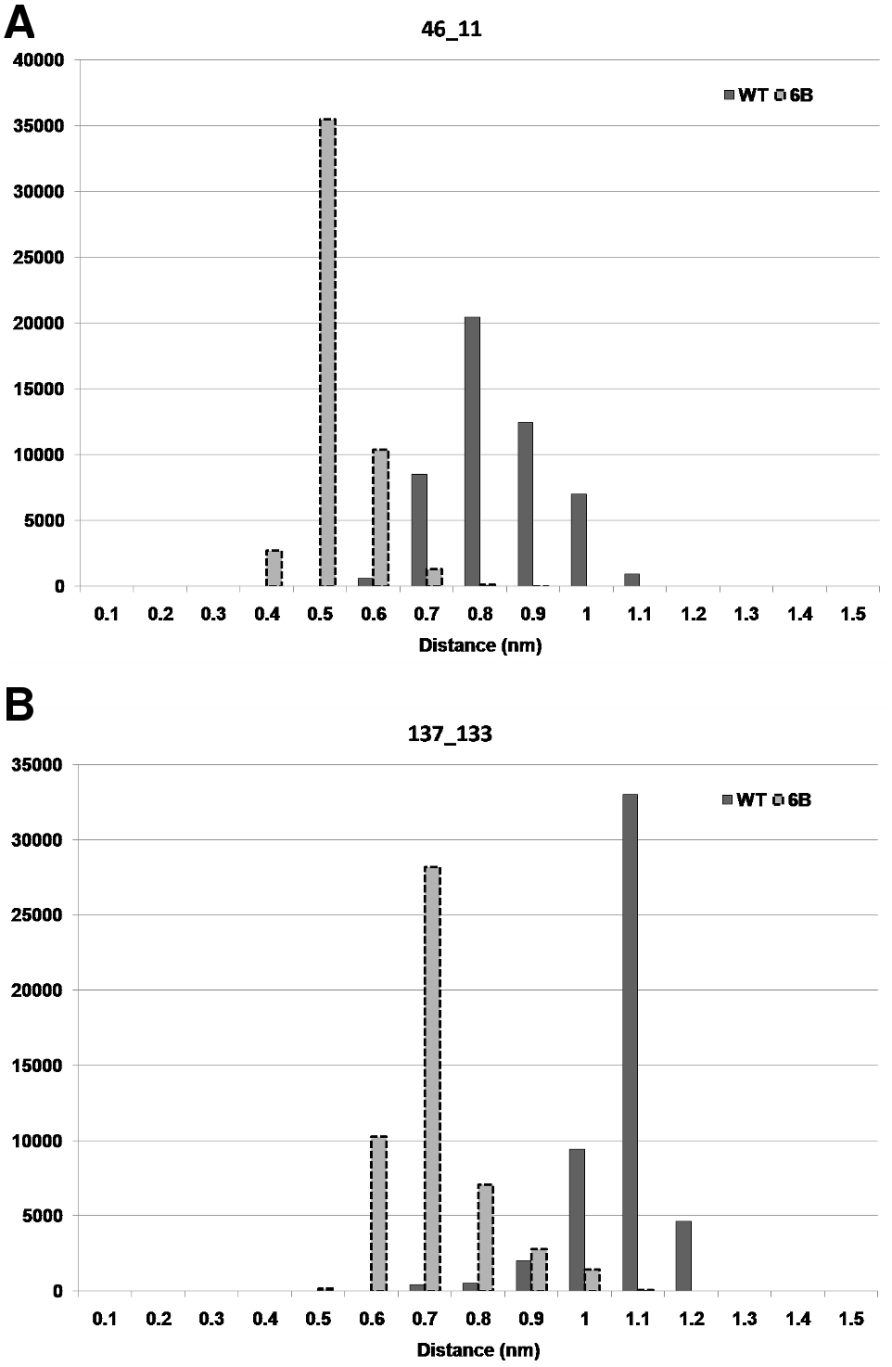
Distribution of distance between Cα atoms. Frequency plot of number of frames against the distance between the Cα atoms of residues: A) 46-11 and B) 137-133.

First we observed the distance between the Cα atoms of the residues involved in the new contacts formed in the mutant 6B as compared to the WT, for the 100 ns trajectory. As shown in Figure 10A the distance between Cα atoms of residue 46 and 11 was lower for a large number of MD structures in mutant 6B than WT. The average value of this distance was 0.79 nm for WT and 0.47 nm for mutant 6B. A similar observation can also be made for contact between residues 137 and 33 (Figure 10B). The distances in this case were 1.02 nm for WT and 0.66 nm for 6B. The frequency distributions of structural snapshots of simulations for 100 ns trajectory, as a function of distances for all the new contacts showing considerable shift in the distance distribution in 6B mutant, are shown in Figure S3. Twelve out of the twenty new contacts show a significant shift towards a lower distance between the residues (two sample KS test p-value<0.001). The shift in the distance distribution towards lower distances suggests that these contacts lead to compactness in the protein in these regions. All the new contacts that show stability during MD simulation have either one or both the residues belonging to a loop. Since the majority of these contacts are long-range contacts, it suggests that the mutant molecule has become more compact/rigid compared to the WT.

#### Root Mean Square Fluctuation (RMSF)

**Figure 11:**
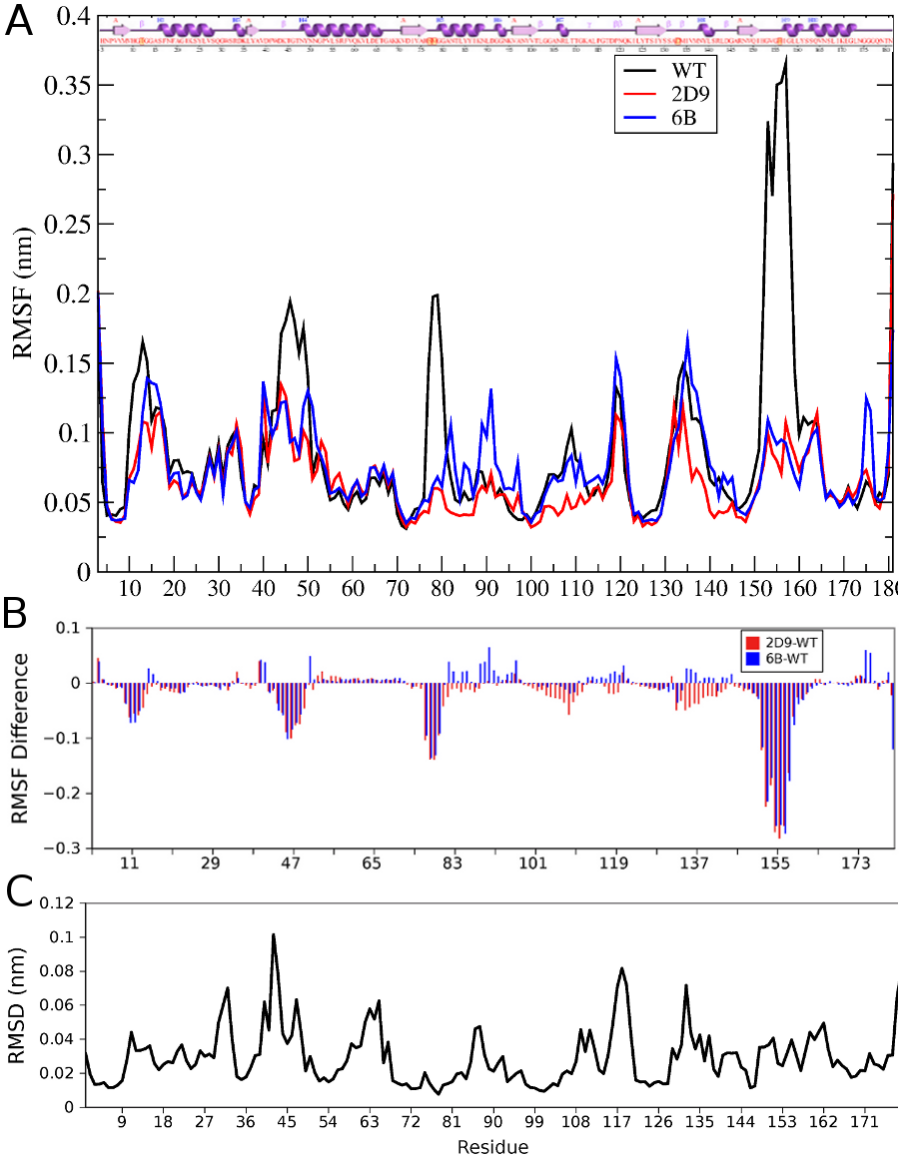
Flexibility analysis of WT and mutants. A) The RMSF values of all residues in WT, 2D9 and 6B from MD simulations. B) The difference in the RMSF values of the mutant 2D9 (Red bars) and 6B (Blue bars) and the WT. C) Residue wise RMSD obtained after aligning the seven structures of Lipase A.

Residue specific fluctuations in the protein are observed using RMSF. Flexible regions show higher values of RMSF as compared to the stable regions. Using 20 ns simulation of 6B mutant it was earlier [36] concluded that the mutations cause rigidity in the structure, particularly in the active site region and this leads to the higher activity of the mutant at higher temperatures. Here we show that this observation also holds for longer simulation times (100 ns at 300 K), but the reduction in flexibility of the molecule is not restricted to just one mutant (shown for both 2D9 and 6B in Figure 11A), and is distributed throughout the structure. It is interesting to note that the 2D9 mutant, which includes six of the twelve mutations present in 6B mutant, shows reduction in flexibility in the same regions as 6B, in the RMSF analysis. This indicates the effect of these particular regions of the proteins in conferring overall stability to the mutants.

Comparison of WT RMSF profile with the two mutants highlights that the reduction in the flexibility is specific to few regions. Figure 11B gives the difference in RMSF values of 2D9 (Red bars) and 6B (Blue Bars) as compared to WT. There are five major regions where the RMSF shows reduction in case of the mutants. These regions comprise residue - 1) 10–18; 2) 43–49; 3) 77–81; 4) 152–159, and 5) 3 and 181. Four of these regions contain residues primarily in loops. The reduction in RMSF indicates the stabilization of these loops and consequently the increased rigidity in the active site. There is also a reduction of RMSF in the termini of the molecule (Region 5). There are seven new contacts that have formed in 6B that involve one of these regions. Thus the contacts made in these regions stabilize these regions and reduce the flexibility. Both rigidity of active site, loop regions and stabilization of terminal regions are in agreement with the results that were obtained by network analysis.

This result was further corroborated by the principal component analysis of the WT and 6B MD trajectories at 300 K. The first ten PCs accounted for 71.7% and 57.0% of the total variance in the dynamics of WT and mutant 6B respectively. The values for the trace of the covariance matrix were 2.03 and 1.18 for WT and 6B respectively. Higher value in case of WT suggests higher flexibility associated with WT. This flexibility is contributed mainly by motions in the loops around the active site (77–80, 152–159) as well as away from the active site (45–49, 10–14) and also the termini (3,181). The motion described by the first PC in WT essentially represents the motion of these residues (Figure S4). However in the case of the mutant, the regions mentioned above show low flexibility. This may be due to the new contacts made in these regions (31-3, 45-13, 46-11, 81-48, 155-134, 181-113 and 181-115). Also the residues (I12, G14, A15, I151 and G153) belonging to these regions show an increase in their clustering coefficient suggesting increase in the cliquishness in network.

The RMSF values were also compared to the residue wise RMSD obtained after aligning the WT and six mutant structures (Figure 11C). The residue wise RMSD plot expectedly shows similarity to the RMSF plot obtained for the mutants. This suggests that the fluctuations observed in MD simulations of the mutants are similar to the deviations observed between X-ray crystal structures of different variants.

The structural factors attributed to induce enhanced stability of thermostable proteins include hydrogen bonds, solvent accessible surface area (SASA), salt bridge and secondary structure content [4], [7], [8], [38]. Some of these important factors were studied in the MD simulations to compare the WT and 2D9 (3D2B) and 6B mutants (3QMM).

#### Solvent Accessible Surface Area (SASA)

**Figure 12:**
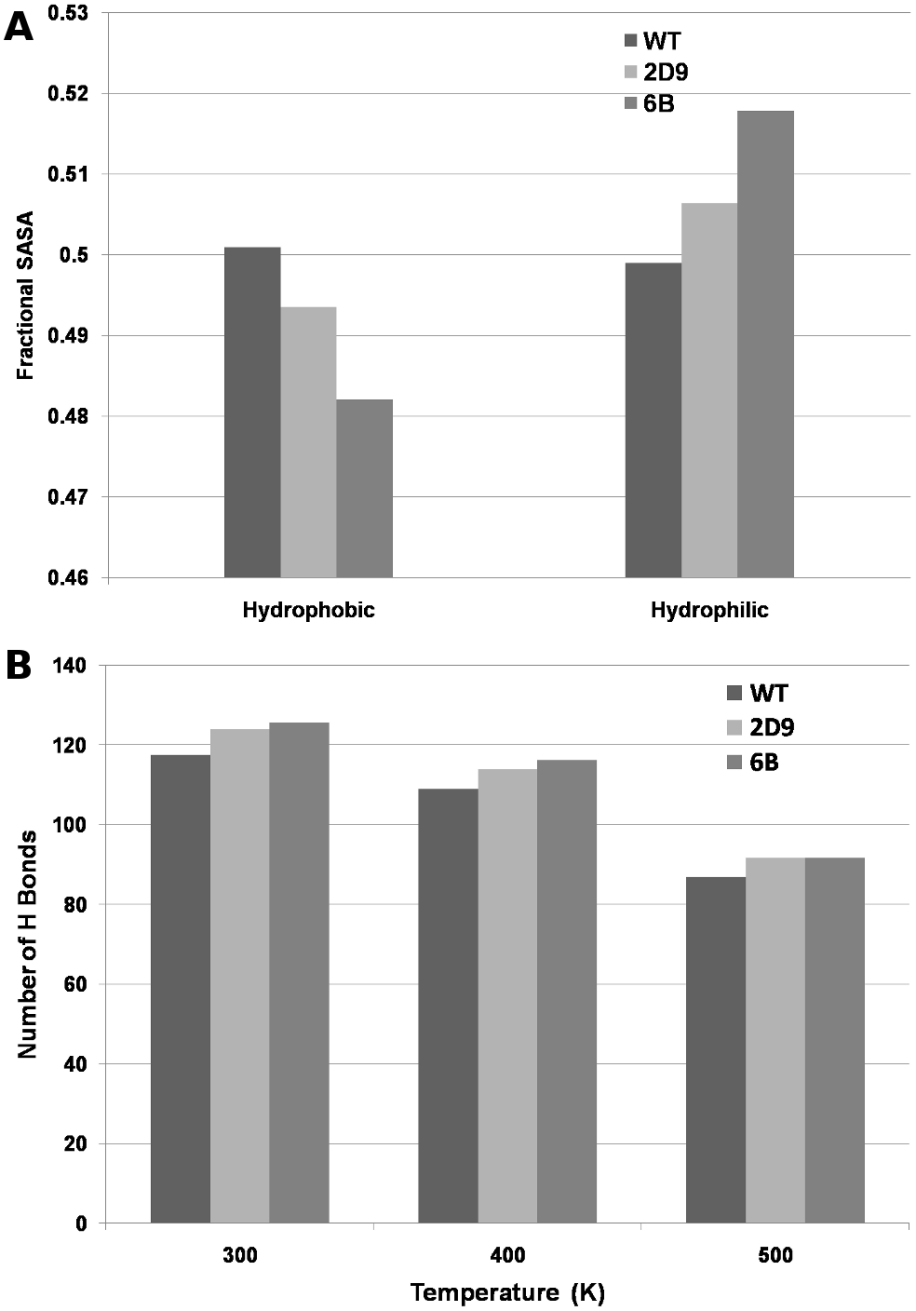
SASA and Average number of Hydrogen bonds per frame. A) Average Fractional Hydrophilic and Hydrophobic SASA values of the frames of MD trajectories for WT, 2D9 and 6B. B) Average number of Hydrogen Bonds per frame for WT, 2D9 and 6B at 300 K, 400 K and 500 K.

Solvent accessible surface area represents compactness of a protein structure [18]. Increase in the polar SASA and simultaneous decrease in the non-polar SASA has been implicated in the increased stability of the thermo stable proteins [8]. We calculated the SASA values for the 100 ns trajectory of MD simulation for WT, 2D9 mutant and 6B mutant. The average total solvent accessible surface is significantly lower (p<0.05) in case of mutated structure 6B (91.04 nm^2^) as compared to the WT structure (95.28 nm^2^). The fractional polar and non-polar SASA values, calculated from each frame of the 100 ns trajectory were significantly different (Wilcoxon ranksum test p<0.05) between the WT and the mutant 6B (Figure 12A). Figure 12A also shows the average fractional SASA values for the 2D9 mutant. The values for 2D9 were in intermediate range between WT and 6B. Thus, our simulation results show increase in the polar exposed surface area and decrease in non-polar exposed surface area, along with an overall reduction in the total SASA in the mutant. This indicates that the mutant structures become more compact in comparison to the wild type. However, it may be noted that the fractional polar and non polar surface area for the WT and 6B were found similar (∼0.4 and ∼0.6) for the crystallographic structure [28]. This may be due to the fact that the crystal structure represents only one of the several conformations that the structures might be sampling in the MD simulations.

#### Hydrogen Bonds

The average number of hydrogen bonds per frame of the 100 ns MD simulations for WT, and thermostable proteins 2D9 and 6B were calculated. The average number of hydrogen bonds per frame is higher (125.6, Wilcoxon Ranksum Test, p<0.05) in 6B mutant as compared to the WT (117.5– see Figure 12B 300 K). The average number of hydrogen bonds per frame in case of 2D9 mutant was found to be 123.9. Higher number of hydrogen bonds have been previously related to the increased thermostability of thermophilic proteins [7]–[9]. Figure 12B shows that the average number of hydrogen bonds per frame increases with increasing thermostability in the 300 K simulations.

#### Salt Bridges

Electrostatic interactions, in the form of salt bridges, are known to play important role in the stability, folding and function of proteins [39]. The role of salt bridges in thermostability has been thoroughly studied and has been found to be a major factor contributing to the enhanced stability of the thermophilic proteins [4]. Salt bridges were calculated for the MD simulation trajectories of WT and 6B mutant at 300 K, as described in Methods. A total of twenty-one salt bridges were found in WT and twenty-nine in the mutant trajectory. Further, number of frames having the center of mass of the positively charged atoms in basic side chain and negatively charged atoms in the acidic side chains at a distance less than 5 Å was calculated for each trajectory. All the salt bridges along with this number have been listed in Table S9. As seen from this table, the number of salt bridges has increased in the mutant, which may contribute to the overall stability of the mutant.

### High Temperature Molecular Dynamics Simulations

Since protein denaturation occurs typically in microsecond time scale [40], it is difficult to capture the unfolding of protein at normal temperatures using molecular dynamic processes. In order to study denaturation process in WT and mutant proteins within the feasible time limits, much higher temperatures are used. MD simulations at higher temperatures have been performed previously to study the thermostability in Xylanases [5]. It has been shown earlier [41] that higher temperature accelerates the unfolding process without changing the unfolding pathway, at least in the initial stages of unfolding. Working on this assumption, we have analyzed the unfolding of WT and the mutant Lipases 2D9 and 6B at higher temperatures.

The optimum temperature of activity for the 6B mutant was reported as 65°C in comparison to WT protein, which is active at 35°C [28]. The 6B mutant exhibited increased activity at all temperatures, as compared to WT or any other mutant. The major reason for this phenomenon was argued to be the rigidity of the active site using MD simulation and Fluorescence study [36]. However, the factors leading to this rigidity, and contributions of the entire protein to this phenomenon, were not discussed. Here we attempt to shed some light on these questions by performing MD simulations of WT, 2D9 and 6B mutants at higher temperatures of 400 K and 500 K for 30 ns each.

#### Root Mean Square Deviations (RMSD)

**Figure 13:**
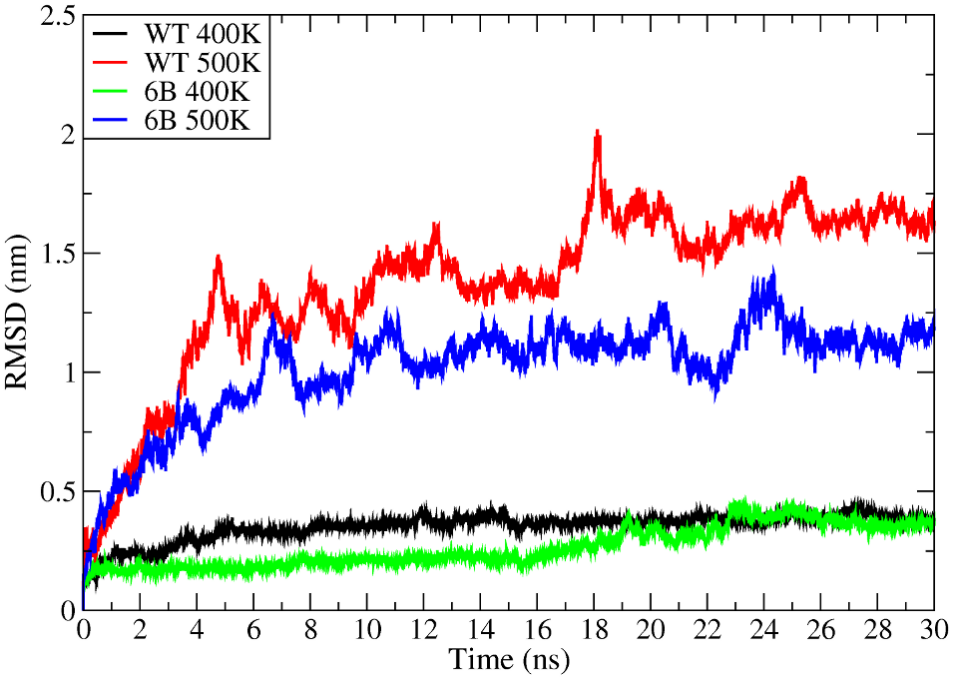
RMSD of Cα atoms. Root Mean Square Deviations of Cα atoms for WT and 6B at 400 K (Black and Green respectively) and 500 K (Red and Blue respectively).

Average RMSD for WT and 6B mutant for 300 K, 400 K and 500 K are shown in Table 3. At 300 K, the RMSD of the WT and 6B do not show much difference. However there is a clear trend in RMSD values at higher temperatures. RMSD values at 400 K and 500 K show a decrease with the increase in thermostability. Figure 13 shows RMSD traces for four high temperature MD simulations for the WT and 6B structures at 400 K and 500 K for 30 ns corroborating the results mentioned above.

**Table 3:** Average RMSD values (nm) at different temperatures.

Temperature	WT	6B
**300**	0.1628	0.1624
**400** a	0.3506	0.2643
**500** a	1.3711	1.0144

a WT values in all comparisons are significantly higher than 6B at p<0.01.

#### Secondary Structure Content

Total number of residues occurring in regular secondary structure is an indicator of the stability of the protein. Figure S5 shows the number of residues in different secondary structures in each frame of the MD simulations at different temperatures. At all three temperatures simulated here, the average number of residues occurring in regular secondary structures are higher in mutant 6B compared to WT (Text S1).

#### Hydrogen Bonds

The average number of hydrogen bonds in the mutant 6B is significantly higher (Wilcoxon ranksum test p<0.01) than that in WT at both 400 K and 500 K (Figure 12B 400 K and 500 K). As hydrogen bonds play an important role in the maintenance of the secondary as well as tertiary structure of the molecule, mutant proteins should show higher resilience towards denaturation at higher temperatures. Also at 400 K the average number of hydrogen bonds per frame increases with increasing thermostability as shown in Figure 12B for both 2D9 and 6B proteins.

#### Solvent Accessible Surface Area

Denaturation is marked by an increase in solvent exposed surface area as water solvates the unfolding molecule. Figure S6 shows that the average fractional polar SASA value at 400 K is higher in case of mutants 6B and 2D9. This shows that the extent of unfolding in the case of WT is higher at 400 K, as the non-polar regions start getting exposed to the solvent. At 500 K, WT and both the mutants show a higher fractional non-polar SASA.

## Discussion

The Lipase A protein and its carefully selected *in vitro* evolved thermostable and functional mutants [26]–[28] offer a convenient system to study the structural changes that enhance thermostability without inducing much changes in their overall three-dimensional conformation. We have used the Lipase A protein and all the six thermostable mutants to uncover the general features underlying evolution of increased thermostability in the absence of significant changes in the three-dimensional structures by - a) performing a comparative coarse-grained protein contact network-based study to extract the contact changes and their influence in network parameters, b) finding the structure stabilizing effects of the new contacts, c) analyzing the changes in the community structures, between the wild-type and mutant structures, and d) supplementing the network-based results with room temperature and high temperature molecular dynamics study of the proteins. Even though these different approaches give us important information on how the thermostability of the mutants is achieved due to mutations in the protein, we have aimed at arriving at a consolidated view by connecting these different approaches.

The study on contact changes uncovered establishment of important stabilizing contacts due to mutations, which gives important clues to thermostability. The total number of contacts formed is higher than the number of contacts lost suggesting an increase in the compactness of the protein. Important contacts were found to be made near termini and active site residues, which stabilize these regions, and hence add to the thermostability of proteins. Both these observations were confirmed by the reduced RMSF values of the termini and the active site regions indicating that the formation of these contacts has actually reduced the flexibility of these regions. The results obtained by analysis of network parameters also corroborate these results. The residues near the active site show increase in the clustering coefficient, which further suggests the enhancement of rigidity caused by the formation of new contacts.

The community structure analysis clearly shows reorganization of the membership of specific nodes in different communities occurring due to mutation, even when the overall structure shows negligible difference. This indicates changes in communication among the functional and allosteric residues in the whole protein. The establishment of more contacts can also yield a larger number of communities by fragmenting the existing ones, as seen in our analysis. All mutants show fragmentation of the first community of the WT into two different communities where a few residues have become part of a larger Community 4. As shown in the RMSF and RMSF difference plots, the residues (152–159) in community 1 and 4 in WT show high fluctuations, but in mutant 6B these residues now belong to a larger community (Community 4) and exhibit much reduced fluctuations. The expansion of the core region beta sheets into a larger, single community by incorporating a large part of the loop residues is a clear pointer to increased stability of the protein. The community structure analysis, in conjunction with dynamic simulations, strongly indicates features relevant to the establishment of structural stability in mutants. A similar result is obtained from the analysis of residue-wise Betweenness Centrality in mutants. Contact analysis also unveils certain important contacts (e.g P114 to K88) that play crucial role in the overall stability of the protein, but have not been noted in previous structural analysis

In recent years, network-based approaches have been used extensively to understand the structure function relationship of proteins [42]. However, our combined coarse-grained network and molecular-level dynamical modeling approach is an unusual way to analyze small conformational changes in the proteins through the loss or gain of contacts, and relating it to its stability and function. Our PCN analysis has shown that the gain of contacts can lead to the regional stability (termini and loop stabilizations), thereby increasing the overall stability of the protein. Such synergy between the ordered and disordered regions for functional versatility in proteins is increasingly being observed in different systems [43]. Importantly, these small conformational changes are not only restricted to the sites of mutation, but throughout the protein, and thereby, contributing to the thermostability of the protein in a concerted manner. Thus, it becomes important to consider the small changes occurring throughout the proteins, in order to explain the structural and functional aspects of mutations.

Surprisingly, certain new contacts and important structural factors leading to stability of the protein become apparent when dynamics is associated to the network analysis, in comparison to static crystal structures. This is noticeable in the analysis of the SASA where the average hydrophilic SASA is higher in case of thermostable mutants but was not evident from the static crystal structures. Also, other results obtained by the MD simulations, including number of hydrogen bonds and number of salt bridges, indicate towards the higher stability of the mutants. This suggests that the dynamics of the protein also plays a crucial role in defining a quality as important as thermostability. Thus the combined network and dynamics based analysis of protein structures gives novel insight into the enhanced stability of the thermostable mutants of the *Bacillus subtilis* Lipase A. One interesting direction that this approach can take is to construct the PCNs from the ensemble of MD simulations, and study the motion of those important residues identified from the various network based analysis of the WT and mutant structures.

This mesoscopic-network approach and fine-grained molecular dynamics approach can be a convenient and useful scheme to elucidate the small but important conformational changes among the structures where altered allosteric communication pathways resulting from ligand/drug binding and mutations, underlie changes in functions without significant changes in the overall network structure.

## Methods

### Dataset used

Lipase A is an extracellular lipase that catalyses both hydrolysis and synthesis of long-chain triacylglycerols. It is a 19.34 kDa protein consisting of 181 amino acids and is much smaller than other lipases. It is a globular protein resembling α/β hydrolase fold. It consists of six β-strands in a parallel β-sheet surrounded by two α-helices on one side and three on the other [34] (Figure 1). *Bacillus subtilis* Lipase A structures used in the analysis are listed in Table 1. All the protein structure coordinate files were taken from Protein Data Bank.(www.pdb.org) [44]. The “directed evolution” method used to obtain the thermostable mutants offered a set of six mutants with increasing thermostability with each successive mutant retaining the earlier mutations in them.

### Protein Structure Alignment

Multiple structure alignment of the proteins was performed using Protein structure comparison service Fold at European Bioinformatics Institute (http://www.ebi.ac.uk/msd-srv/ssm) [45].

### Protein Contact Network Construction

PCNs were constructed for all seven protein structures as described in [30], [33]. Each Cα atom of the residues in the protein is considered as a node and the spatial proximity between them defines the link. In this coarse grained representation any two nodes are considered to be linked by an edge if the distance between them is <0.7 nm. This cut-off has been widely used for constructing Cα based protein contact network and provides a reasonable estimate of the interacting residue pairs in the first interaction shell [46]. However there are other cut-offs also that have been used when considering all atom network models [47].

In this way a protein structure consisting of N amino acid residues can be represented as adjacency matrix (A) such that
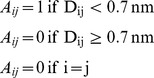
where D_ij_ is the distance between Cα atom of i^th^ and j^th^ residue.

The PCNs were also constructed with two other realistic thresholds - 0.65 nm and 0.80 nm, and the analysis repeated for the WT and mutant 6B. The absolute number of contacts made or lost obviously depends on the threshold used for constructing the PCNs, but the ratios of the two are very similar and statistically insignificant (Wilcoxon test with p-value 0.7 and 0.3 when 0.70 nm is compared to 0.65 nm and 0.80 nm, respectively). Additionally, in both cases, like the PCNs with 0.70 nm cut-off, most changes in contacts in the mutant are at the allosteric sites and do not involve the active site residues.

### Network Parameters

The network parameters (Degree, Betweenness Centrality, Closeness Centrality, Clustering Coefficient, and Shortest Paths) were calculated using igraph package [48] of R [49]. The details of these network parameters are provided in Text S1.

### Community Calculation

Communities in the PCNs of WT and the mutant structures were calculated using the “igraph” package. Fast greedy algorithm proposed by Clauset et. al. [50] was used for calculating the best split of communities.

### Molecular Dynamics Simulations

The X-ray structures of WT (PDB ID 1I6W) and 6B mutant (PDB ID 3QMM) were used as starting models for MD simulations. MD simulations were performed using GROMACS software package [51]. Both proteins were simulated at 300 K, 400 K and 500 K for 100 ns, 30 ns and 30 ns respectively. The simulations were done by soaking starting structures in explicit solvent using TIP4P water model and OPLSAA force field. Periodic Boundary conditions were used with minimum distance between the solute and the box of 0.9 nm. The systems were neutralized by replacing appropriate number of water molecules by Cl- ions. We used only counter ions and not the explicit salt conditions as these are more important only for highly charged systems [52]. The systems were subjected to energy minimization using steepest descent method for 50000 steps or till the maximum force on all atoms was less than 1000.0 kJ/mol/nm. Energy minimization was followed by equilibration first in an NVT ensemble for 200 ps and then in an NPT ensemble for 200 ps. After the systems were equilibrated, the production run was performed for 100 ns at 300 K and 30 ns at 400 K and 500 K. The equations of motion were integrated with a time step of 2fs and the coordinates were saved every 1000 time steps (2ps) resulting in total of 50001 frames for 100 ns simulations and 15001 frames for 30 ns simulations. Particle Mesh Ewald (PME) summation [53] was used for calculating electrostatic interactions. All-bonds were constrained using LINCS algorithm [54]. A constant temperature was maintained by weak coupling to modified Berendson thermostat [55] and Parinello Rahman pressure coupling [56] was used to maintain a constant pressure during simulations.

2D9 mutant (PDB id 3D2B) was also simulated for 100 ns at 300 K, and for 30 ns at 400 K and 500 K, following the same protocol and parameters as mentioned above for WT and 6B.

### Analysis of MD Simulation Trajectories

All the analysis was done using the frames from the production run (50001 for 300 K and 15001 for 400 K and 500 K). RMSD, RMSF, Hydrogen Bonds and SASA were calculated using *g_rms*, *g_rmsf*, *g_hbond* and *g_sas* functions of GROMACS respectively. The secondary structure content in each frame of the trajectories was calculated using the *do_dssp* function of GROMACS which in turn uses DSSP [57] to assign secondary structures.

To extract the essential motions from the trajectory, principal component analysis [58] was performed using the *g_covar* function of GROMACS. The eigen values and the eigen vectors obtained as a result of PCA were analyzed using the *g_anaeig* function of GROMACS.

Salt bridges were calculated using the Salt Bridge Plugin in the VMD [59]. The distance cutoff between the oxygen atom of the acidic side chain and the nitrogen atom of the basic side chain was selected as 4 Å. The distance between the centre of masses of the positive charges atoms in the basic side chain and the negatively charged atoms in the acidic side chains were also calculated for every fifth frame of the trajectory (total 10002 frames).

All the programs for the construction of adjacency matrix from PDB files and contact analysis were written in MATLAB R2007b (www.mathworks.com). Adjacency matrix calculated using MATLAB was used as input for network parameter calculation in igraph package of R. Two-sample Kolmogorov-Smirnov Test was performed in R and Wilcoxon ranksum test was performed in MATLAB.

The protein structures were visualized and the figures were generated using PyMOL.

Solvent accessible surface area (SASA) for the crystal structures of WT and the mutants was calculated using the POPS program [60]. The SASA values calculated for the crystal structures showed no consistent trend with respect to compactness of the wild type and mutant structures (Table S10). However the crystal structure represents only one of the several conformations that the structures might be sampling in actual solvated environment. Therefore, SASA values were calculated for the 100 ns trajectory of molecular dynamics simulation (50000 frames) for WT and 6B mutant.

## Supporting Information

Figure S1 **Multiple structural alignment of the six mutants and WT.** WT (Green), DM (Magenta), TM (Cyan), 1–17A4 (Yellow), 2D9 (Deep Salmon), 4D3 (Gray) and 6B (Lime).
(TIF)

Figure S2 **Ring graph representations of the contacts lost and made in five mutants a) 1T4M b) 1T2N c) 3D2A d) 3D2B e) 3D2C.**
(TIF)

Figure S3 **Distance distribution of all the new contacts formed in the mutant 6B, that show considerable change in** **the MD simulation.**
(TIF)

Figure S4 **RMSF of Cα atoms after projection of WT (Black) and 6B (Red) 300 K simulation trajectories on their respective first principal components.**
(TIF)

Figure S5 **Number of residues in different secondary structures in A) WT at 400 K B) 6B at 400 K C) WT at 500 K and D) 6B at 500 K.**
(TIF)

Figure S6 **Average Fractional SASA values for WT, 2D9 and 6B at 400 K and 500 K.**
(TIF)

Table S1 **Average Network Parameters for the PCNs.**
(PDF)

Table S2 **Residues with highest BC values.**
(PDF)

Table S3 **Ten residues in each mutant showing maximum difference in the betweenness centrality.**
(PDF)

Table S4 **Residues with highest clustering coefficient.**
(PDF)

Table S5 **Ten residues in each mutant showing maximum difference in the clustering coefficient.**
(PDF)

Table S6 **Contacts made in all the mutants.**
(PDF)

Table S7 **Contacts lost in all the mutants.**
(PDF)

Table S8 **Community membership of the nodes in all the PCNs.**
(PDF)

Table S9 **List of all the salt bridges in WT and mutant 6B.**
(PDF)

Table S10 **SASA values for crystal structures.**
(PDF)

Text S1 **Supplementary Methods and Results.**
(PDF)
