# Supplementary figures and images for: Thermostability of In Vitro Evolved Bacillus subtilis Lipase A: A Network and Dynamics Perspective
Source: PLoS One. 2014 Aug 14;9(8):e102856. doi: 10.1371/journal.pone.0102856 (PMC4133394; doi:10.1371/journal.pone.0102856)

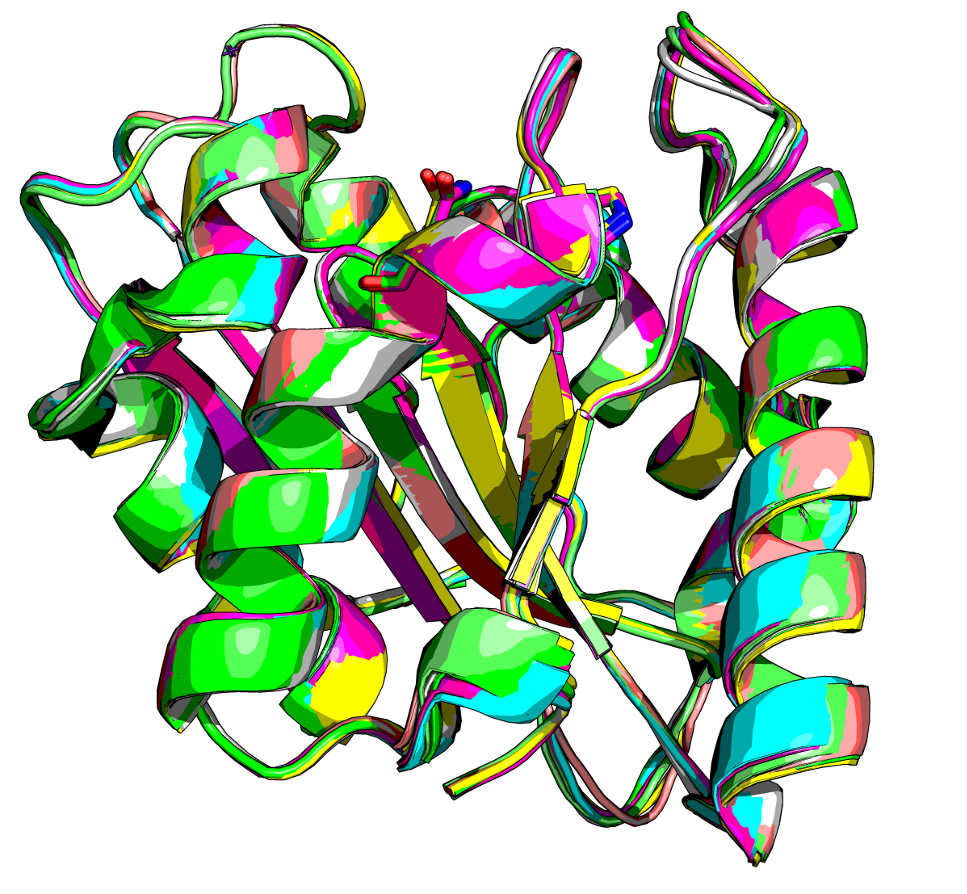

Supplement: Figure S1 — Multiple structural alignment of the six mutants and WT. WT (Green), DM (Magenta), TM (Cyan), 1–17A4 (Yellow), 2D9 (Deep Salmon), 4D3 (Gray) and 6B (Lime). (TIF) [file pone.0102856.s001.tif]

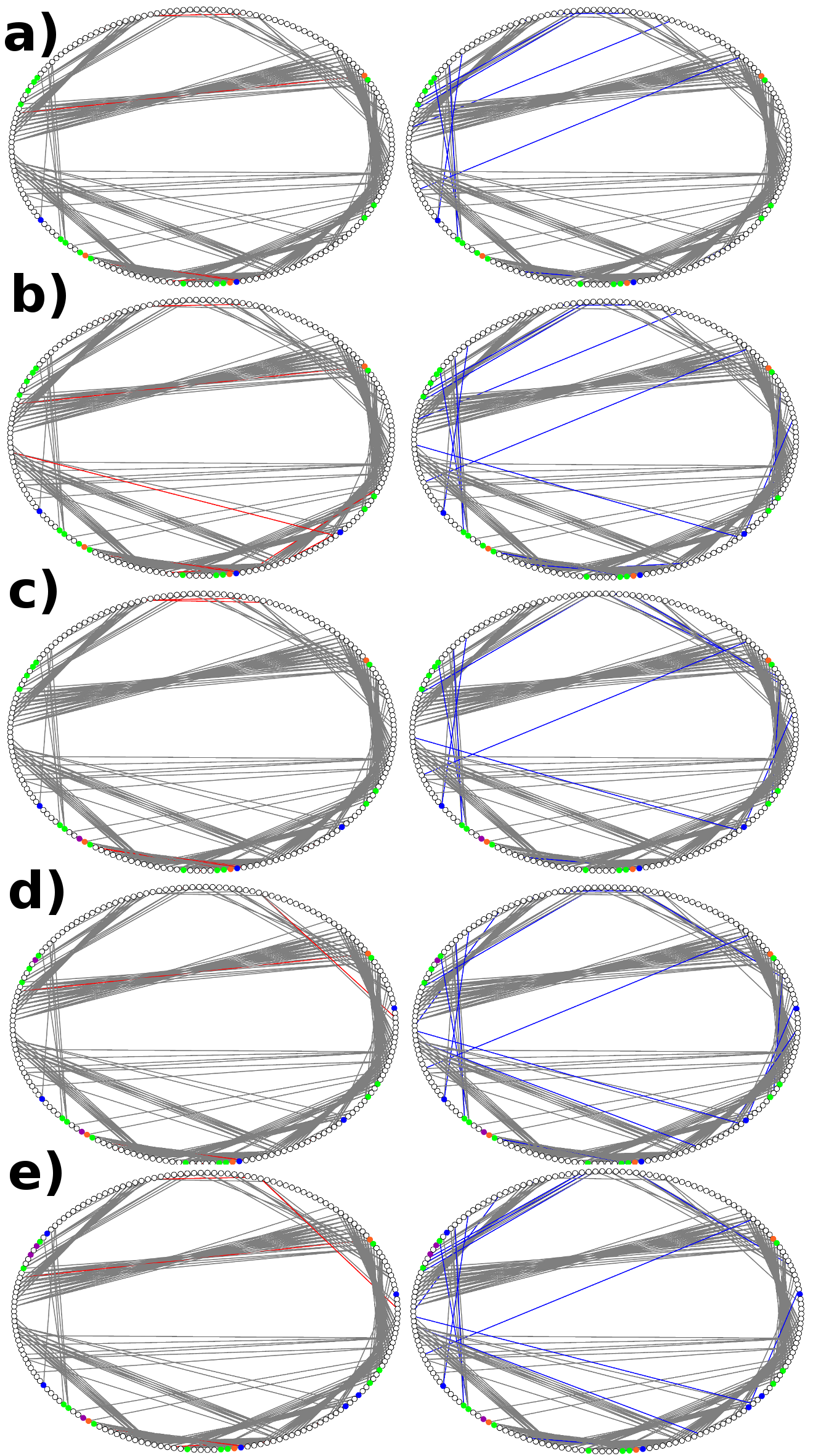

Supplement: Figure S2 — Ring graph representations of the contacts lost and made in five mutants a) 1T4M b) 1T2N c) 3D2A d) 3D2B e) 3D2C. (TIF) [file pone.0102856.s002.tif]

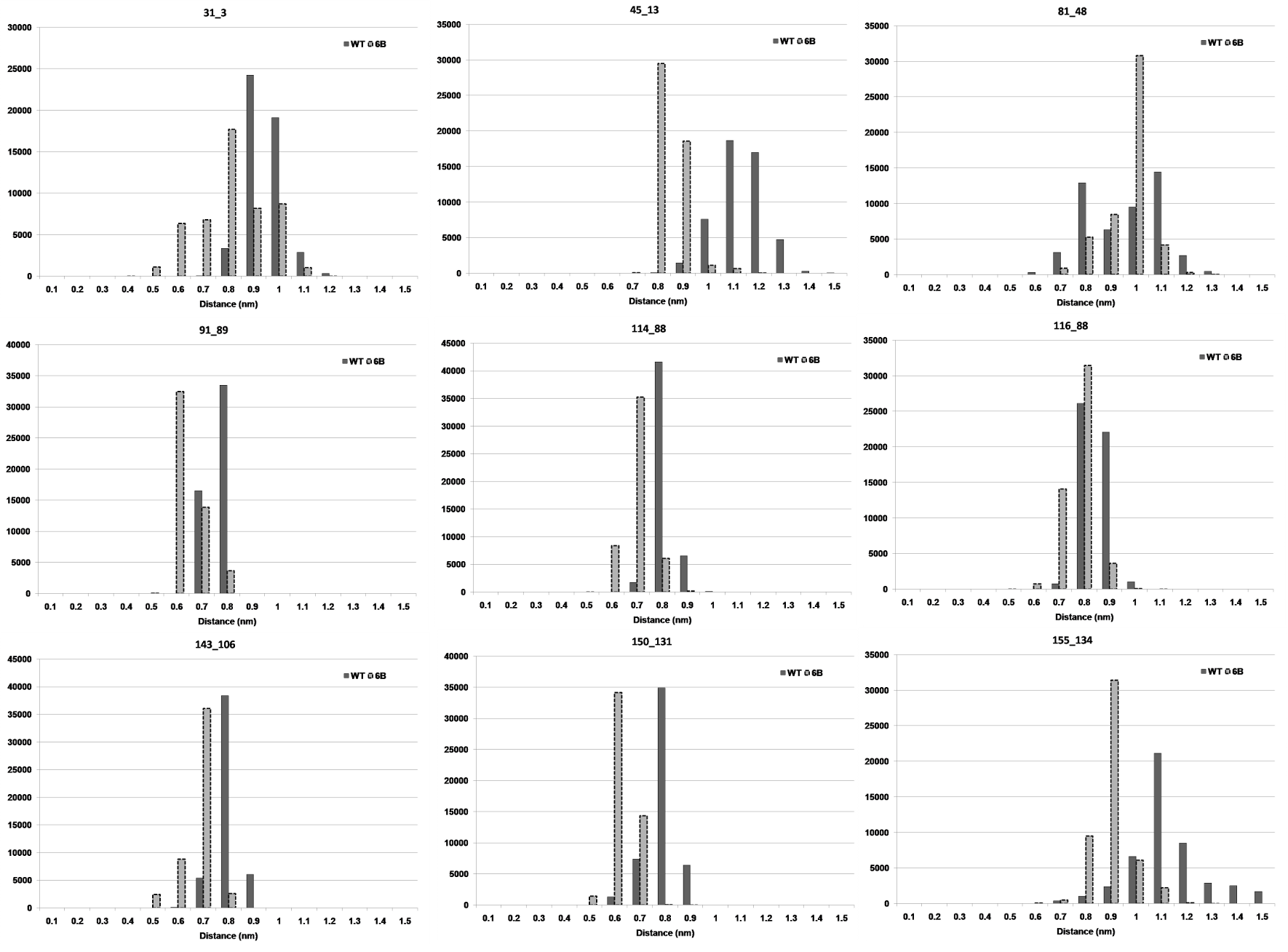

Supplement: Figure S3 — Distance distribution of all the new contacts formed in the mutant 6B, that show considerable change in the MD simulation. (TIF) [file pone.0102856.s003.tif]

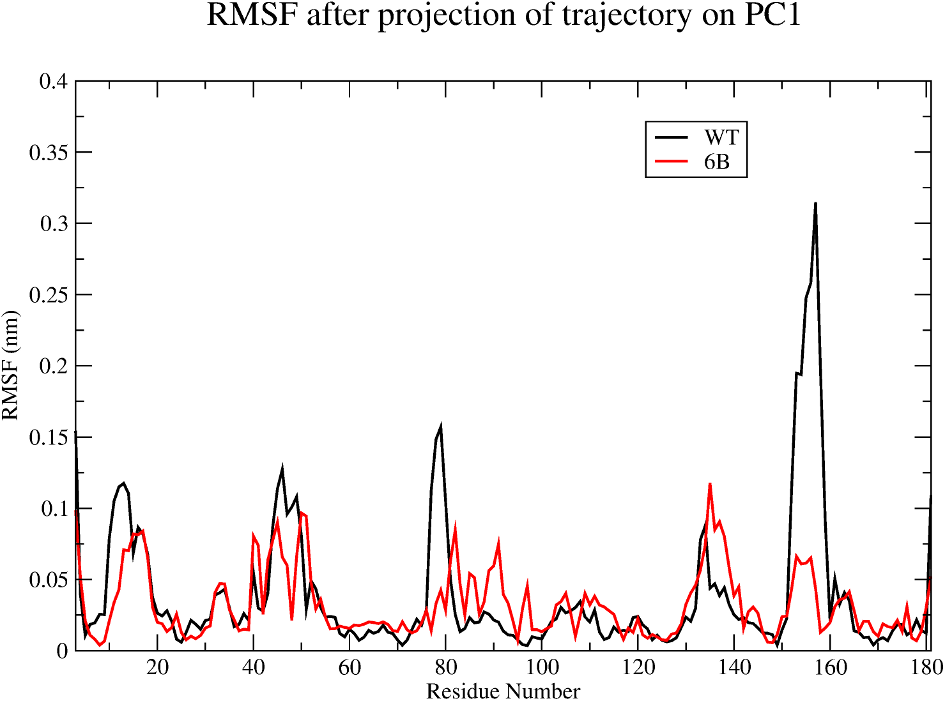

Supplement: Figure S4 — RMSF of Cα atoms after projection of WT (Black) and 6B (Red) 300 K simulation trajectories on their respective first principal components. (TIF) [file pone.0102856.s004.tif]

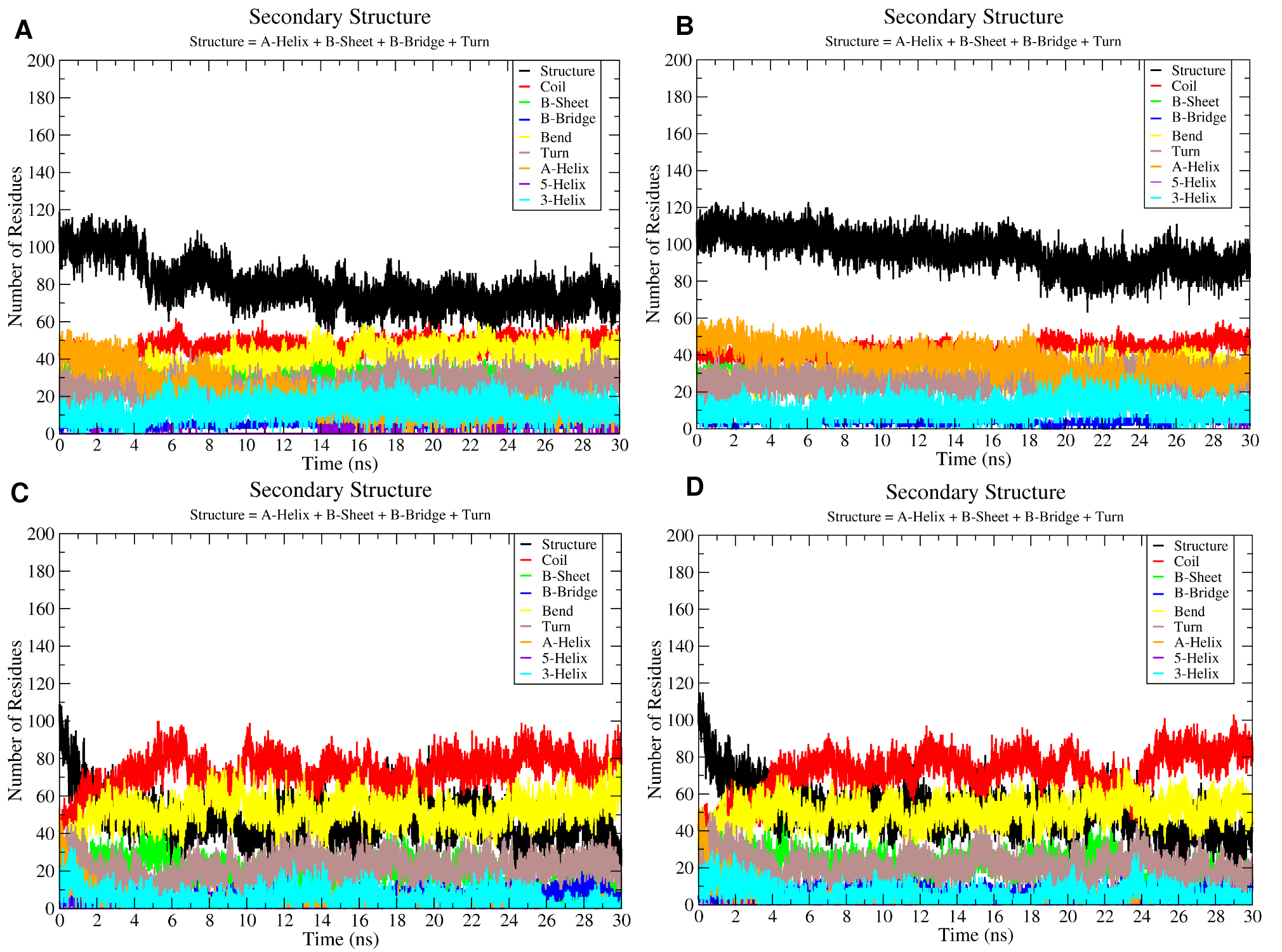

Supplement: Figure S5 — Number of residues in different secondary structures in A) WT at 400 K B) 6B at 400 K C) WT at 500 K and D) 6B at 500 K. (TIF) [file pone.0102856.s005.tif]
