# Supplementary material for: Thermostability of In Vitro Evolved Bacillus subtilis Lipase A: A Network and Dynamics Perspective
Source: PLoS One. 2014 Aug 14;9(8):e102856. doi: 10.1371/journal.pone.0102856 (PMC4133394; doi:10.1371/journal.pone.0102856)
Supplement: Table S1 — Average Network Parameters for the PCNs. (PDF) [file pone.0102856.s007.pdf]

Table S1 Average Network Parameters for the PCNs

Protein	No of Edges	Degree	Betweenness	Closeness	Avg. Clustering Coefficient	Avg. Shortest Path
WT	738	8.25	0.0183	0.240	0.539	4.24
DM	741	8.28	0.0180	0.242	0.528	4.20
TM	746	8.36	0.0180	0.242	0.532	4.19
1-17A4	741	8.28	0.0184	0.240	0.531	4.25
2D9	751	8.40	0.0180	0.234	0.527	4.18
4D3	748	8.36	0.0180	0.244	0.528	4.17
6B	746	8.34	0.0180	0.242	0.523	4.19
