# Supplementary material for: Thermostability of In Vitro Evolved Bacillus subtilis Lipase A: A Network and Dynamics Perspective
Source: PLoS One. 2014 Aug 14;9(8):e102856. doi: 10.1371/journal.pone.0102856 (PMC4133394; doi:10.1371/journal.pone.0102856)
Supplement: Table S2 — Residues with highest BC values. (PDF) [file pone.0102856.s008.pdf]

Table S2 Residues with highest BC values. Residues with high BC values in all the PCNs are colored as Yellow. Residues with high BC in all mutants are Cyan

1I6W	z-score	1T4M	z-score	1T2N	z-score	3D2A	z-score	3D2B	z-score	3D2C	z-score	3QMM	z-score
100	3.41	7	3.70	7	3.90	100	3.48	100	3.47	100	3.75	100	3.55
102	3.19	100	3.50	100	3.38	72	3.21	72	3.05	75	3.49	102	3.38
39	3.07	102	3.21	102	3.36	102	3.09	102	2.97	102	3.13	159	3.03
98	2.98	159	2.97	159	2.92	159	2.76	39	2.75	72	2.98	72	3.01
159	2.78	72	2.74	72	2.79	101	2.60	103	2.68	159	2.67	39	2.89
103	2.51	103	2.52					159	2.67			103	2.54
