# Supplementary material for: Thermostability of In Vitro Evolved Bacillus subtilis Lipase A: A Network and Dynamics Perspective
Source: PLoS One. 2014 Aug 14;9(8):e102856. doi: 10.1371/journal.pone.0102856 (PMC4133394; doi:10.1371/journal.pone.0102856)
Supplement: Table S3 — Ten residues in each mutant showing maximum difference in the betweenness centrality. (PDF) [file pone.0102856.s009.pdf]

Table S3 Ten residues in each mutant showing maximum difference (increase or decrease) in the betweenness centrality, as compared to WT. Residues common to all the mutant PCNs are shown in yellow and those common in three or more, out of six mutants are shown in cyan

Positive	1T4M-1I6W		1T2N-1I6W		3D2A-1I6W		3D2B-1I6W		3D2C-1I6W		3QMM-1I6W
59	0.042	59	0.042	18	0.023	18	0.022	83	0.038	88	0.017
7	0.038	7	0.040	81	0.022	173	0.016	75	0.034	173	0.017
25	0.015	18	0.023	72	0.020	88	0.015	18	0.023	81	0.015
173	0.015	173	0.017	173	0.016	72	0.015	173	0.015	72	0.014
72	0.010	146	0.017	63	0.015	25	0.013	72	0.015	25	0.014
18	0.010	88	0.015	70	0.015	3	0.012	88	0.014	3	0.013
166	0.010	25	0.015	51	0.014	38	0.012	25	0.013	143	0.010
38	0.009	72	0.010	10	0.014	114	0.009	52	0.013	48	0.010
11	0.009	114	0.010	25	0.014	83	0.009	3	0.012	11	0.010
41	0.008	166	0.008	83	0.013	123	0.009	38	0.012	166	0.009
Negative											
39	-0.037	39	-0.037	39	-0.055	98	-0.018	98	-0.022	98	-0.022
19	-0.018	98	-0.019	58	-0.029	19	-0.016	55	-0.019	36	-0.014
55	-0.017	130	-0.019	16	-0.024	22	-0.014	19	-0.018	82	-0.012
98	-0.015	19	-0.018	19	-0.019	16	-0.013	39	-0.017	141	-0.011
22	-0.014	16	-0.017	76	-0.018	122	-0.012	92	-0.017	22	-0.011
16	-0.012	103	-0.017	22	-0.015	124	-0.011	16	-0.017	124	-0.011
58	-0.011	55	-0.017	55	-0.013	116	-0.010	22	-0.015	87	-0.010
37	-0.010	125	-0.016	98	-0.012	39	-0.009	80	-0.013	122	-0.010
70	-0.010	22	-0.014	122	-0.012	36	-0.009	82	-0.013	78	-0.010
32	-0.008	58	-0.012	78	-0.011	23	-0.009	96	-0.011	160	-0.008
