# Supplementary material for: Thermostability of In Vitro Evolved Bacillus subtilis Lipase A: A Network and Dynamics Perspective
Source: PLoS One. 2014 Aug 14;9(8):e102856. doi: 10.1371/journal.pone.0102856 (PMC4133394; doi:10.1371/journal.pone.0102856)
Supplement: Table S4 — Residues with highest clustering coefficient. (PDF) [file pone.0102856.s010.pdf]

Table S4 Residues with highest clustering coefficient. Residues having high CC in all the PCNs are shown in yellow. Residues common only in mutants are shown in Cyan.

1I6W	z-score	1T4M	z-score	1T2N	z-score	3D2A		3D2B		3D2C		3QMM	
3	3.15	3	3.38	3	3.31	3	3.32	34	3.41	34	3.41	34	3.53
34	3.15	181	3.38	89	3.31	34	3.32	120	3.41	89	3.41	120	3.53
89	3.15	49	2.66	120	3.31	89	3.32	49	2.69	49	2.69	49	2.79
120	3.15	89	2.66	49	2.60	120	3.32	89	2.45	139	2.69	89	2.54
181	3.15	34	2.19	174	2.37	153	2.14	174	2.45	174	2.44	174	2.54
49	2.46	120	2.19	34	2.13			90	2.04	120	2.20	153	2.30
139	2.46	153	2.19	153	2.13					181	2.20	28	2.05
174	2.24											29	2.05
												65	2.05
												139	2.05
												157	2.05
												164	2.05
