# Supplementary material for: Thermostability of In Vitro Evolved Bacillus subtilis Lipase A: A Network and Dynamics Perspective
Source: PLoS One. 2014 Aug 14;9(8):e102856. doi: 10.1371/journal.pone.0102856 (PMC4133394; doi:10.1371/journal.pone.0102856)
Supplement: Table S5 — Ten residues in each mutant showing maximum difference in the clustering coefficient. (PDF) [file pone.0102856.s011.pdf]

Table S5: Ten residues in each mutant showing maximum difference (increase or decrease) in the clustering coefficient, as compared to WT. Residues common to all the mutants PCNs are shown in yellow and those common in three or more, out of six mutants are shown in cyan.

Positive	1T4M-1I6W		1T2N-1I6W		3D2A-1I6W		3D2B-1I6W		3D2C-1I6W		3QMM-1I6W
135	0.200	135	0.200	135	0.200	135	0.200	42	0.167	153	0.100
42	0.167	42	0.167	39	0.151	90	0.095	12	0.143	135	0.100
55	0.115	130	0.127	55	0.115	150	0.076	55	0.115	12	0.095
153	0.100	55	0.115	58	0.115	136	0.071	135	0.100	44	0.067
12	0.095	153	0.100	153	0.100	108	0.067	153	0.067	117	0.067
136	0.071	12	0.095	106	0.075	153	0.067	151	0.067	151	0.067
151	0.067	179	0.091	136	0.071	14	0.067	111	0.057	115	0.060
115	0.048	106	0.075	108	0.067	91	0.067	92	0.056	111	0.057
37	0.048	136	0.071	14	0.067	117	0.067	106	0.053	90	0.048
39	0.044	108	0.067	151	0.067	131	0.060	15	0.048	94	0.048
Negative											
121	-0.100	18	-0.105	49	-0.100	25	-0.115	25	-0.115	25	-0.115
139	-0.100	25	-0.115	139	-0.100	54	-0.115	173	-0.115	173	-0.115
18	-0.105	54	-0.115	18	-0.105	173	-0.115	83	-0.132	89	-0.133
173	-0.107	173	-0.115	132	-0.107	89	-0.133	45	-0.167	137	-0.133
25	-0.115	59	-0.143	173	-0.107	88	-0.167	88	-0.167	45	-0.167
54	-0.115	34	-0.167	25	-0.115	31	-0.190	120	-0.167	31	-0.190
59	-0.143	45	-0.167	88	-0.167	121	-0.200	181	-0.167	88	-0.194
34	-0.167	88	-0.167	121	-0.200	114	-0.200	31	-0.190	121	-0.200
45	-0.167	114	-0.200	114	-0.200	181	-0.333	114	-0.200	181	-0.300
120	-0.167	181	-0.500	181	-0.333	3	-0.667	3	-0.667	3	-0.667
