# Supplementary material for: Thermostability of In Vitro Evolved Bacillus subtilis Lipase A: A Network and Dynamics Perspective
Source: PLoS One. 2014 Aug 14;9(8):e102856. doi: 10.1371/journal.pone.0102856 (PMC4133394; doi:10.1371/journal.pone.0102856)
Supplement: Table S6 — Contacts made in all the mutants. (PDF) [file pone.0102856.s012.pdf]

Table S6 Contacts made in all the mutants

1T4M		1T2N		3D2A		3D2B		3D2C		3QMM	
		18	10	18	10	18	10	18	10		
						31	3	31	3	31	3
38	36					38	36	38	36		
								44	14		
44	41	44	41					44	41		
45	13	45	13					45	13	45	13
46	11	46	11	46	11	46	11	46	11	46	11
				53	49						
54	41	54	41			54	41			54	41
59	7	59	7								
				81	48					81	48
				83	52	83	52	83	52		
								83	75		
						91	89			91	89
		92	88			92	88				
94	91	94	91								
		107	81	107	81	107	81				
		114	88	114	88	114	88	114	88	114	88
										116	88
						118	93			118	93
123	114										
137	133	137	133	137	133	137	133	137	133	137	133
137	134	137	134	137	134	137	134				
						138	130				
										143	106
		146	124								
150	131	150	131	150	131			150	131	150	131
		155	134			155	134			155	134
160	18	160	18	160	18	160	18	160	18		
161	22	161	22	161	22	161	22	161	22	161	22
166	25	166	25	166	25	166	25	166	25	166	25
173	72	173	72	173	72	173	72	173	72	173	72
										179	122
						179	123	179	123	179	123
								181	113	181	113
		181	115	181	115	181	115			181	115
