# Supplementary material for: Thermostability of In Vitro Evolved Bacillus subtilis Lipase A: A Network and Dynamics Perspective
Source: PLoS One. 2014 Aug 14;9(8):e102856. doi: 10.1371/journal.pone.0102856 (PMC4133394; doi:10.1371/journal.pone.0102856)
Supplement: Table S7 — Contacts lost in all the mutants. (PDF) [file pone.0102856.s013.pdf]

Table S7 Contacts lost in all the mutants

1T4M		1T2N		3D2A		3D2B		3D2C		3QMM	
36	32	36	32								
47	43	47	43	47	43	47	43	47	43	47	43
										54	50
55	39	55	39	55	39			55	39		
				58	39						
				76	74						
77	10	77	10			77	10	77	10	77	10
90	85										
						91	56				
								92	56		
		111	106	111	106			111	106	111	106
113	111										
122	119			122	119	122	119	122	119	122	119
				122	120	122	120			122	120
		125	113								
		130	103								
										140	136
141	137	141	137	141	137	141	137			141	137
143	127	143	127					143	127		
						152	150				
153	130	153	130	153	130					153	130
153	131	153	131	153	131	153	131	153	131	153	131
				154	133						
176	173			176	173						
										177	98
										178	145
		179	115								
