# Supplementary material for: Thermostability of In Vitro Evolved Bacillus subtilis Lipase A: A Network and Dynamics Perspective
Source: PLoS One. 2014 Aug 14;9(8):e102856. doi: 10.1371/journal.pone.0102856 (PMC4133394; doi:10.1371/journal.pone.0102856)
Supplement: Table S8 — Community membership of the nodes in all the PCNs. (PDF) [file pone.0102856.s014.pdf]

**Table S8 Community membership of the nodes in all the PCNs**

| Community | 1I6W | 1T4M | 1T2N | 3D2A | 3D2B | 3D2C | 3QMM |
|---|---|---|---|---|---|---|---|
| **Community 1** | 21-34<br>157-176 | 162-176 | 162-176 | 162-176 | 162-176 | 162-176 | 21-30 |
| **Community 2** | 12-20<br>40-69 | 12-34<br>40-46<br>160-161 | 12-34<br>40-46<br>160-161 | 86-91 | 103<br>129-141<br>150-159 | 47-69 | 163-176 |
| **Community 3** | 85-94<br>114-123 | 47-70<br>86-97<br>117-121 | 47-69<br>82-94<br>114-123 | 76,77<br>79-81<br>83-85<br>103-110<br>129-141<br>150-159 | 41-69<br>76-91<br>104-110 | 76,77<br>79-91<br>103-110<br>129-141<br>150-159 | 63-69 |
| **Community 4** | 106-113<br>124-156<br>177-181 | 98<br>112-116<br>122-139<br>142-159<br>177-181 | 111-113<br>124-140<br>142-159<br>177-181 | 12<br>40-69<br>78,82 | 10-34<br>40<br>160,161 | 3-9<br>35-39<br>70-75<br>92-102<br>111-128<br>142-149<br>177-181 | 102,103<br>111-114<br>127-162<br>177-181 |
| **Community 5** | 3-11<br>35-39<br>70-84<br>95-105 | 3-11<br>35-39<br>71-85<br>99-111<br>140-141 | 3-11<br>35-39<br>70-81<br>95-110<br>141 | 10,11<br>13-34<br>160,161 | 3-9<br>35-39<br>70-75<br>92-102<br>111-128<br>142-149<br>177-181 | 10-34<br>40-46<br>78<br>160,161 | 10-20<br>40-62<br>76-91<br>104-110 |
| **Community 6** | | | | 3-9<br>35-39<br>70-75<br>92-128<br>142-149<br>177-181 | | | 3-9<br>31-39<br>70-75<br>92-101<br>115-125<br>179 |
