# Supplementary material for: Thermostability of In Vitro Evolved Bacillus subtilis Lipase A: A Network and Dynamics Perspective
Source: PLoS One. 2014 Aug 14;9(8):e102856. doi: 10.1371/journal.pone.0102856 (PMC4133394; doi:10.1371/journal.pone.0102856)
Supplement: Table S9 — List of all the salt bridges in WT and mutant 6B. (PDF) [file pone.0102856.s015.pdf]

**Table S9** List of all the salt bridges in WT and mutant 6B

| WT(1I6W) | No. Frames(out of 10002) with COM of charges <=5Å | 6B(3QMM) | No. Frames(out 0f 10002) with COM of charges <=5Å |
|---|---|---|---|
| ASP118-LYS69 | 383 | ASP111-ARG107 | 8245 |
| ASP118-LYS70 | 9622 | ASP111-ARG142 | 208 |
| ASP133-HIS152 | 14 | ASP111-LYS112 | 813 |
| ASP133-HIS156 | 6687 | ASP111-LYS88 | 10 |
| ASP144-ARG107 | 7725 | ASP118-LYS69 | 471 |
| ASP144-ARG142 | 2463 | ASP118-LYS70 | 7868 |
| ASP144-LYS112 | 1857 | ASP132-HIS152 | 4 |
| ASP34-ARG33 | 383 | ASP133-HIS156 | 7092 |
| ASP34-HIS3 | 1 | ASP144-ARG107 | 1274 |
| ASP34-LYS35 | 5454 | ASP144-ARG142 | 2261 |
| ASP40-ARG57 | 0 | ASP144-LYS112 | 8633 |
| ASP40-HIS10 | 15 | ASP34-ARG33 | 218 |
| ASP40-LYS44 | 26 | ASP34-LYS35 | 8430 |
| ASP64-LYS61 | 599 | ASP40-ARG57 | 14 |
| ASP72-HIS3 | 0 | ASP40-HIS10 | 90 |
| ASP91-LYS95 | 9667 | ASP40-LYS44 | 3513 |
| GLU171-ARG147 | 618 | ASP40-LYS61 | 16 |
| GLU171-LYS170 | 2886 | ASP43-HIS10 | 0 |
| GLU65-ARG33 | 0 | ASP64-LYS61 | 338 |
| GLU65-LYS35 | 2 | ASP72-HIS3 | 23 |
| GLU65-LYS61 | 5545 | ASP72-LYS70 | 581 |
| | | ASP91-ARG57 | 0 |
| | | ASP91-LYS95 | 4165 |
| | | GLU171-ARG147 | 3490 |
| | | GLU171-LYS170 | 2026 |
| | | GLU20-ARG33 | 6556 |
| | | GLU65-LYS35 | 261 |
| | | GLU65-LYS61 | 4634 |
| | | GLU20-LYS23 | 8755 |
