# Supplementary material for: Thermostability of In Vitro Evolved Bacillus subtilis Lipase A: A Network and Dynamics Perspective
Source: PLoS One. 2014 Aug 14;9(8):e102856. doi: 10.1371/journal.pone.0102856 (PMC4133394; doi:10.1371/journal.pone.0102856)
Supplement: Table S10 — SASA values for crystal structures. (PDF) [file pone.0102856.s016.pdf]

Table S10: SASA values for crystal structures

Structure	SASA (nm^2)
1I6W	84.84
1T4M	120.97
1T2N	105.34
3D2A	92.44
3D2B	80.33
3D2C	78.68
3QMM	81.44
