# Supplementary material for: Thermostability of In Vitro Evolved Bacillus subtilis Lipase A: A Network and Dynamics Perspective
Source: PLoS One. 2014 Aug 14;9(8):e102856. doi: 10.1371/journal.pone.0102856 (PMC4133394; doi:10.1371/journal.pone.0102856)
Supplement: Text S1 — Supplementary Methods and Results. (PDF) [file pone.0102856.s017.pdf]

SUPPLEMENTARY METHODS

Network Parameters Analysed

Degree and Degree distribution

Degree of a node (k) is defined as the total number of nodes that it is directly connected to. Degree distribution represents the fraction of nodes P(k) having a particular degree k. It is an important parameter as it depends largely on the topology of the network.

Clustering Coefficient

Clustering Coefficient measures the cliquishness of the neighbourhood of the node. It is defined as the ratio of the number of edges among the nearest neighbor of the node to the total number of edges possible among them.

$$C_i = 2 \times n / k_i(k_i - 1)$$

Where, k_i =number of neighbours of node i
n = number of contacts amongst k_i neighbours

Shortest Path and Characteristic Path Length (Average Shortest Path)

Shortest Path between a pair of nodes is defined as the smallest number of links that need to be traversed in order to reach from one node to another. An average of shortest paths between all the pairs of nodes in a network is defined as Characteristic Path Length (CPL). CPL is a global property that determines the typical separation between two nodes in a network

$$CPL = \frac{1}{n(n-1)} \sum_{i,j} d(v_i, v_j)$$

Where, n is number of vertices, $d(v_i,v_j)$ is the shortest path between vertices v_i and v_j

Betweenness Centrality

Betweenness of a node can be roughly defined as the number of shortest paths going through the node. Thus, Betweenness Centrality B_x of a node x is

$$B_x = \sum_{\substack{i,j \epsilon V \\ i \neq x \neq j}} \frac{\sigma_{ixj}}{\sigma_{ij}}$$

where, σ_{ixj} is total number of shortest paths between i and j that pass through x
σ_{ij} is total number of shortest paths between i and j
The residues with significantly high betweenness centrality are determined by defining a z score

$$Z_x = \frac{B_x - \overline{B}}{\sigma_B}$$

Where, $\overline{B}$ is the average Betweenness Centrality and σ_B is the standard deviation. The residues with $Z_x \geq 2.5$ are considered to have high betweenness centrality.

Closeness Centrality

The Closeness Centrality of a residue is defined as the inverse of the sum of Shortest Paths from that residue to all other residues in network.

$$\boldsymbol{C_x} = \frac{\boldsymbol{n-1}}{\sum_{i \neq x} \boldsymbol{d(i,x)}}$$

SUPPLEMENTARY RESULTS

Correspondence between the contacts made/lost and network parameters

The local effects of gain or loss of the contacts in mutants are reflected in the network parameters at the residue-level. In the thermostable mutants having L114P mutation – i) the gain of contact shows as increase in degree of K88 in all the mutants, ii) K88 is among the top ten residues with highest increase in the betweenness centrality in four out of five mutants, iii) residues 114 and 88 are among the top ten residues with highest decrease in the clustering coefficient in all mutants, and iv) clustering coefficient of residue N181 also decreases in all the mutants. This decrease might be due to addition of a new neighbor without the simultaneous addition of edges of it with the other neighbors. The residues in the nearby region also show changes in their clustering coefficients, which might be a consequence of the change in the contact pattern.

Secondary Structure Content analysis in high temperature simulations

Among the secondary structures, higher number of residues is in α helices in the mutant. Also average number of residues in loops increases rapidly at higher temperatures in both structures, but the rise is more rapid in case of WT than in the mutant. These results indicate that the secondary structures in the thermostable mutant are maintained more stably compared to the WT.

To get an overall structural comparison of the WT and the mutant at high-temperature MD simulations, we analyzed the secondary structures of the snapshots taken from the high temperature simulations of WT and 6B mutant at 400K. We took six snapshots at equal intervals from the 30 ns trajectory of the proteins at 400K. The secondary structure of each frame was determined using DSSP. The analysis shows that the helix αA (residue 20-29) melts into a smaller 3_{10} helix in WT by 5ns, and melts completely by30ns, whereas in mutant 6B it remains stable (although it decreases in size). Similarly helix αC (residue 79-88) also melts within 5 ns in to a smaller 3_{10} helix, whereas in mutant it remains stable till about 20ns and then melts into a combination of regular and 3_{10} helix. A similar observation can also be made for the helix αF (residues 163-173). β sheet at the core of the molecule remains mostly stable in both WT and mutant. There are two new contacts (166-25 and 161-22) that have been made between the helices αA and αF. Also one of the new contacts is formed between helix αF and strand β5 (173-72). New Contact between residues 114 and 88 connects helix αC to the longest loop in the structure. Thus the snapshot analysis interestingly indicates towards the enhanced stability in the regions that show new contacts being made in the structure.
Thus, it is clear that the mutant structure, in spite of showing very little conformational change, compared to the WT in crystal structures, shows important differences in the dynamical parameters. This also confirms higher stability and resilience of the mutant in solution at higher temperatures compared to the WT.
